# Genome-wide analysis of the laccase gene family in tossa jute (*Corchorus olitorius*): insights into stem development, lignification, and responses to abiotic stress

**DOI:** 10.3389/fpls.2025.1568674

**Published:** 2025-05-09

**Authors:** Deepak Kumar Jha, Subhadarshini Parida, Seema Pradhan, Nrisingha Dey, Shuvobrata Majumder

**Affiliations:** ^1^ Department of Plant & Microbial Biotechnology, BRIC-Institute of Life Sciences (ILS), NALCO Square, Bhubaneswar, Odisha, India; ^2^ BRIC-Regional Centre for Biotechnology, Faridabad, India

**Keywords:** jute, laccase, whole genome search, microRNA, abiotic stress, lignin pathway

## Abstract

Tossa jute (*Corchorus olitorius*) dominates global jute cultivation but has a high lignin content (13-14%) in its fibres, making them coarse and limiting their industrial applications. Reducing the lignin content requires a deeper understanding of the lignification process and the associated genes. Laccase (EC 1.10.3.2) is a key enzyme in the final step of lignin biosynthesis. A genome-wide analysis of the 361 Mb *C. olitorius* genome identified 46 laccase genes (*ColLAC*s) from a total of 28,479 genes. *In-silico* analysis revealed that *ColLAC* genes are distributed across seven chromosomes, encode proteins ranging from 7.98 to 173.99 kDa, with 74 to 1548 amino acids and 10 conserved motifs. Additionally, 48.83% of ColLACs are predicted to be transmembrane proteins. Phylogenetic analysis classified them into eight groups, with GO term assignments suggesting their involvement in lignification. Tissue-specific expression analysis showed that 43.47% of *ColLAC* genes are predominantly expressed in roots, aligning with RNA-seq data. *ColLAC* gene expression varied across the developmental stages, from seedling to fibre harvest, and was influenced by heavy metal copper and abscisic acid (ABA) treatments. This variation correlated with upstream cis-acting elements. *Ath-miR397* target sites were identified in 14 *ColLAC* genes, indicating potential post-transcriptional regulation. Further expression analysis in X-ray-induced *bfs* (bast fibre-shy) mutant tossa jute lines suggested that *ColLAC34* is involved in both lignification and structural development, while *ColLAC22, ColLAC40*, and *ColLAC46* play key roles in lignification. This study presents the first comprehensive genome-wide identification and characterization of the *LAC* gene family in jute. Understanding *ColLAC* functions could facilitate the development of low-lignin jute fibres, meeting the growing industrial demand for high-quality natural fibres of jute.

## 1 Introduction

Lignocellulosic jute (*Corchorus* sp.) fibres have multiple commercial applications industrially and domestically in paper, packaging, and fabrics, producing a variety of sustainable products. The fibres are bast or stem fibres, specifically the phloem tissue, extracted at around 120 days of plant age. Jute plants, valued as the most significant among stem fibre crops in the world, create a soaring global demand for their long, lustrous, strong, biodegradable/organic golden fibres. The only limitation of these fibres is their high lignin content, which makes them rough, rigid, and prone to discoloration upon storage. This makes them unsuitable for applications where softness and smoothness are priorities, and low lignin bast fibres such as flax (*Linum usitatissimum*) or lignin-free flower fibres such as cotton (*Gossypium herbaceum*) are preferred.

Commercially there are only two cultivated jute species and both of them are lignin rich. Lignin content in the fibres of white jute (*Corchorus capsularis*) was found to be 12-13% and that of tossa jute (*C. olitorius*) 13-14% (Majumder et al., 2020a). Economically, there is a persistent and heavy demand for low lignin jute fibres, which necessitates low-lignin fibre production. This can only be achieved by acquiring detailed knowledge of the lignification process and the associated genes in the jute plant system.

Lignin is a complex aromatic polymer that provides structural support, facilitates water and nutrient transport, and contributes to plant defence mechanisms (Dixon and Barros, 2019). It is primarily composed of three monolignol-derived subunits: p-hydroxyphenyl (H) from p-coumaryl alcohol, guaiacyl (G) from coniferyl alcohol, and syringyl (S) from sinapyl alcohol (Boerjan et al., 2003). The lignin biosynthesis pathway is intricate and partially unresolved, with certain enzymatic steps yet to be fully characterized (Goujon et al., 2003). In white jute, the expression of key lignin biosynthesis genes, including *PAL1* (phenylalanine ammonia-lyase; EC 4.3.1.24), *C4H1* (cinnamate 4-hydroxylase; EC 1.14.13.11), *4CL1* (4-coumarate:CoA ligase; EC 6.2.1.12), *CCoAoMT2* (caffeoyl-CoA *O*-methyltransferase; EC 2.1.1.104), *CCR1* (cinnamoyl-CoA reductase; EC 1.2.1.44), *F5H1* (ferulate 5-hydroxylase; EC 1.14.-.-), *COMT1* (caffeic acid *O-*methyltransferase; EC 2.1.1.68), and *CAD7* (cinnamyl alcohol dehydrogenase; EC 1.1.1.195), has been reported by Chakraborty et al. (2015).

In our previous qRT-PCR-based gene expression study, we re-examined the expression of eight key lignin biosynthesis genes—*PAL1, C4H1, 4CL1, CCoAoMT2, CCR1, F5H1, COMT1*, and *CAD7*—in jute phloem tissue at different developmental stages. The analysis spanned from 30 days after sowing (DAS), when lignification began, to fibre harvest at 120 DAS, using ubiquitin1 and beta-tubulin as internal control genes (Parida et al., 2024b). However, information on several crucial genes in the jute lignin pathway, such as peroxidase (*PRX*) and laccase (*LAC*), remains unexplored. Among the lignin biosynthesis pathway genes, a systematic gene family analysis has only been conducted on *CCoAoMT2* (Akhter et al., 2022). This study presents the first comprehensive genome-wide identification and characterization of the *LAC* gene family in tossa jute (*C. olitorius*).

LAC is intrinsic to the final stage of lignin polymerization (Boerjan et al., 2003). The *LAC* gene products comprise a heterogeneous collection of multicopper oxidases capable of facilitating one electron oxidations (Berthet et al., 2012). Multiple investigations have suggested the potential function of *LAC* in lignin polymerisation (Sterjiades et al., 1993). Recently, our group conducted a genome-wide study on this enzyme family in the white jute system and reported the active presence of 34 *CcaLAC* genes (Parida et al., 2024a). Investigations made in the past have reported many *LAC* genes in *Arabidopsis thaliana* (17) (McCaig et al., 2005), *Populus trichocarpa* (49) (Lu et al., 2013), *Gossypium hirsutum* (84) (Balasubramanian et al., 2016) *Linum usitatissimum* (45) (Le Roy et al., 2017), *Punica granatum* (57) (Shi et al., 2023), *Glycine max* (93) (Wang et al., 2019) and *Eucalyptus grandis* (54) (Arcuri et al., 2020) along with their involvement in the lignification process of the respective plants. Knockout experiments of *LAC* in Arabidopsis (Berthet et al., 2011) and cotton (Yang et al., 2024) have confirmed their involvement in lignification, fibre development, and stem structure.

To understand the functionality of *LAC* genes in jute plant development and fibre lignification, detailed analysis of these genes is necessary, and knockout lines could serve as valuable models for such studies. While knockout lines are available for all 17 *LAC* genes in Arabidopsis, similar resources were not available for jute. Creating knockout lines in jute is challenging without first identifying the target genes. As an alternative, X-ray-irradiated mutant jute could help identify key *LAC* genes that are directly involved in growth and development. One such mutant jute variety is the *bfs* (bast fibre-shy) tossa, which lacks the ability of differentially developing secondary phloem fibres (SPF) and secondary xylem (wood), but can still develop fibre with some lignin content (Kundu et al., 2012). It matures earlier, produces significantly fewer bast fibres and wood, and yields lower-quality fibres compared to its wild-type control, JRO632 (Kundu et al., 2012). In this study, we used the *bfs* mutant and its control, JRO632, to examine the expression of different *LAC* genes of *C. olitorius* (*ColLACs*).

The expression of *LAC* genes varies across different plant tissues, as documented in various studies. In Arabidopsis and white jute (*C. capsularis*), most *LAC* genes are predominantly expressed in stem tissue rather than in other tissues like leaves, and roots (Parida et al., 2024a). However, in the case of rice (*Oryza sativa*) during its vegetative growth stage and in Eucalyptus, *LAC* expression was mostly observed in roots (Liu et al., 2017; Arcuri et al., 2020). We used leaves, stem, and root tissues of tossa jute, and specifically examined phloem tissue at different time points in its development, from plantlets (30 DAS) to its fibre harvest stage (120 DAS), for *LAC* gene expression analysis.

Gene expression in plants is regulated by both intrinsic cellular mechanisms and external environmental factors. Pre- and post-transcriptional regulations are key processes that control gene expression. Pre-transcriptional regulation involves upstream cis-acting elements, which play a crucial role in gene expression under environmental stresses, developmental stages, hormonal signalling, and normal plant growth. Various cis-elements, such as MYB, MYC, ABRE, ERE, STRE, TGA, GT1, Sp1, GARE-motif, WUN-motif, GATA-motif, TCT-motif, P-box, W-box, G-box, Box-4, RY-element, and LAMP-element, have been identified as regulators of gene expression in plants (Ain-Ali et al., 2021; Jha et al., 2024). *In-silico* analysis can be used to identify the presence of these cis-elements in the promoter region of a gene, providing insights into its potential expression patterns and functional roles. However, no such study has yet been conducted on the *LAC* gene family in jute.

Post-transcriptional regulation in plants is predominantly controlled by miRNAs, which are small (21-nucleotide), non-coding RNAs that negatively regulate gene expression by targeting specific mRNAs for degradation or translational inhibition (Fang and Wang, 2021). Advanced *in-silico* analysis can predict whether a given mRNA is a potential target of miRNA-mediated post-transcriptional regulation. The miR397 family is well known for targeting *LAC* genes, thereby modulating lignin biosynthesis pathways in plants (Kozomara and Griffiths-Jones, 2011). However, in jute, the specific miRNAs involved in lignin pathway regulation and their role in targeting *LAC* genes remain to be explored. Understanding these regulatory mechanisms in jute will provide valuable insights into lignin biosynthesis and its potential genetic manipulation for fibre quality improvement.

Laccase (*LAC*) genes are known to respond to environmental stresses by increasing or decreasing their expression. They react to abiotic factors like heavy metals, drought, salinity, temperature changes, and oxidative stress, as well as biotic factors such as pathogens, insects, and fungi (Bai et al., 2023). However, *LAC* gene expression patterns in jute, under these stress conditions, remain unknown.

Our aim is to discover the gene family of *ColLAC* in *C. olitorius* and evaluate gene expression in different parts of the plant at various developmental stages. We seek to open new frontiers in identifying the *LAC* genes and their functionality in bast fibre production for subsequent functional investigations.

## 2 Materials and methods

### 2.1 The tossa jute genome sequence

For the *in-silico* analysis, the tossa jute genome sequences were downloaded from the Genome Warehouse (https://ngdc.cncb.ac.cn/gwh/Assembly/20707/show, accessed on December 12, 2023) following the method described by Zhang et al. (2021). The downloaded file contained DNA, mRNA (CDS), and protein sequences.

### 2.2 Plant material

Tossa jute cultivars JRO524, JRO632, and the *bfs* mutant were grown under greenhouse conditions (30 ± 2°C, 80% humidity, with a 13-hour light/11-hour dark cycle). Leaf, stem, and root tissues were collected from JRO632 plants at 60 DAS. Phloem tissue was sampled from JRO524, JRO632, and *bfs* plants at 30, 60, 90, and 120 DAS to assess *ColLAC* gene expression across different developmental stages. Stem samples from JRO524 plants subjected to abiotic stress at 60 DAS were also collected. All tissue samples were stored at -80°C until RNA isolation.

**Figure 1:**
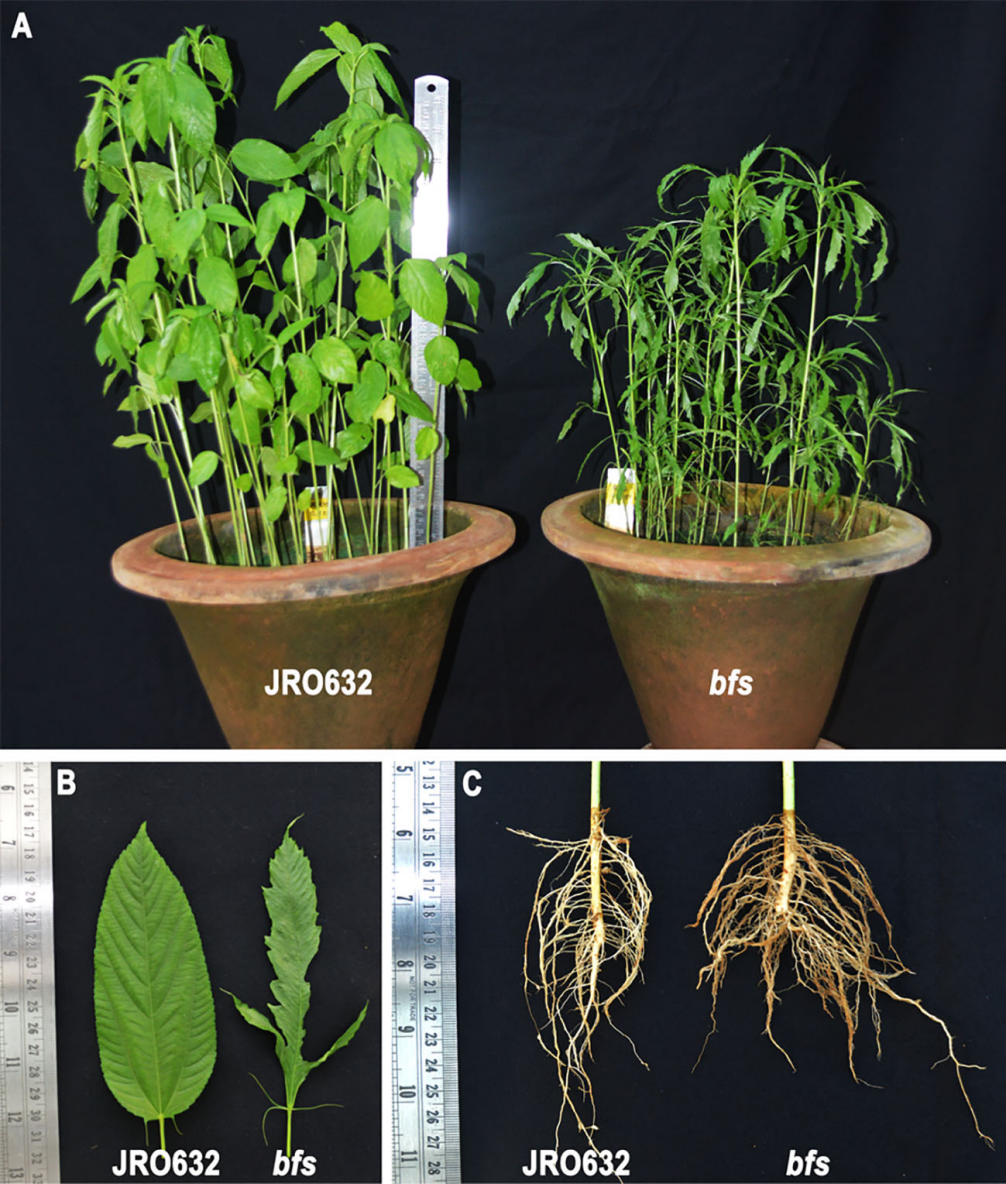
Tossa jute (*Corchorus olitorius*) cultivars JRO632 (control) and *bfs* (X-ray-irradiated mutant) used in the *ColLAC* gene expression study. Comparative images showing differences in **(A)** plant height, **(B)** leaf morphology, and **(C)** root architecture between the control and mutant cultivars.

The *bfs* mutant displays a distinct phenotype characterised by stunted growth (**Figure 1A**), trilobed, split ribbon-like leaves (**Figure 1B**), and a spreading root architecture (**Figure 1C**). These traits make the *bfs* mutant easily distinguishable from the control cultivar, JRO632.

### 2.3 Abiotic stress treatments

Abscisic acid (ABA) stress (0.15 mM) and copper heavy metal stress (0.10 mM CuSO_4_·H_2_O) were applied to healthy, 60-day-old JRO524 plants grown under greenhouse conditions, following the method described by Parida et al. (2024b). Stem tissue samples were harvested at various intervals (0 h, 2 h, 4 h, 8 h, 12 h, and 24 h). For each time point, three separate plants were randomly selected and used as biological replicates in the experiments.

### 2.4 Identification and sequence analysis of *LAC* genes in the tossa jute genome

LAC protein sequences of *A. thaliana* were retrieved from TAIR (Lamesch et al., 2012), *Oryza sativa* sequences were obtained from Oryzabase (Kurata and Yamazaki, 2006), and *LAC* sequences of *Theobroma cacao* were retrieved from the Phytozome database (Goodstein et al., 2012) to create a Hidden Markov Model (HMM) profile. A total of 99 *LAC* gene sequences were used to prepare the HMM profile using the HMMER tool (version 3.2) (Finn et al., 2011). In the HMMER tool, the E-value threshold was set to <1e-5, and the alignment parameters were kept at default settings.

The presence of specific domains (PF00394.19, PF07731.11, and PF07732.12) associated with the LAC protein family in the proposed ColLAC sequences was confirmed utilising NCBI CDD (Marchler-Bauer et al., 2015), SMART (Letunic et al., 2021), or HMM Scan (Finn et al., 2011). All sequence hits with redundancy cut-off values beyond 0.01 were omitted from the analysis, resulting in the inclusion of solely non-redundant hits (Jha et al., 2024). An examination of the physical and chemical properties of the *ColLAC* proteins was performed utilising the ProtParam program on the Expasy website (Wilkins et al., 1999). The transmembrane properties of the *ColLAC* proteins were assessed utilising the TMHMM-2.0 tool (Krogh et al., 2001).

### 2.5 Chromosomal mapping and phylogeny construction

The *ColLAC* genes were physically mapped using the MapInspect program version 1.0, based on their genomic coordinates (https://mapinspect.software.informer.com, accessed on December 10, 2023).

The LAC protein sequences from Arabidopsis (*Arabidopsis thaliana*), white jute (*C. capsularis*), and tossa jute (*C. olitorius*) were selected for phylogenetic analysis. Sequence alignment was performed using the MUSCLE algorithm (Edgar, 2004). Phylogenetic trees were constructed using the maximum likelihood method, employing MEGA software (version 10.0; Kumar et al., 2018). To ensure robustness, the analysis included 1000 bootstrap replications, applying gap deletions and a gamma (G) distribution rate for site variations (Jha et al., 2024).

### 2.6 Motif and gene structure determination

The conserved motifs in *ColLAC* proteins were examined using the MEME suite tool (Bailey et al., 2009). For motif prediction, the analysis was set to identify up to 10 motifs, with the motif width constrained between 6 and 50 amino acids (Chanwala et al., 2023). The Gene Structure Display Server (GSDS) program was used to determine the exon-intron structure of each *ColLAC* gene (Hu et al., 2015).

### 2.7 Gene duplication and synteny analysis

The analysis also included identifying mutations and duplication events in the *ColLAC* gene family. Gene duplication patterns were examined by performing a BLASTp search of *ColLAC* proteins against *LAC* proteins from Arabidopsis, rice, cacao (*T. cacao*), and white jute, using a cutoff e-value of 0.01.

For this analysis, Arabidopsis was chosen as a model plant, rice as a representative monocot with distinct traits from jute, and cacao due to its high genomic similarity to jute, as reported by Sarkar et al. (2017) and Zhang et al. (2021). Sequences with ≥80% similarity were considered for identifying gene duplications (Jha et al., 2021).

To assess evolutionary divergence, synonymous (Ks) and nonsynonymous (Ka) substitution rates were calculated using the PAL2NAL program (Suyama et al., 2006). The synteny relationships were analysed and visualized using TBtools software (Chen et al., 2020).

### 2.8 Cis-element analysis

The upstream regions (~2000 bp) of the *ColLAC* genes were extracted from the *C. olitorius* genome to analyse potential regulatory elements. The PlantCARE database (Rombauts et al., 1999) was used to identify these cis-regulatory elements. The primary focus of this analysis was to identify elements involved in responses to both biotic and abiotic stress, as well as those related to processes like cell cycle regulation, circadian rhythms, development, metabolism, and hormone-mediated signalling pathways.

### 2.9 Gene ontology term annotation

To investigate the functional roles of the *ColLAC* gene family, Gene Ontology (GO) terms were assigned. Protein sequences of *ColLAC* were analysed using BLASTp against the UniProt-SwissProt database (https://www.uniprot.org/uniprotkb?query=reviewed:true, accessed on January 14, 2025) to identify homologous proteins. The corresponding UniProt IDs were then submitted to the Gene Ontology (GO) resource (https://geneontology.org/, accessed on January 14, 2025) for GO term annotation, using *AtLAC* as a reference for functional categorization.

### 2.10 *In-silico* expression analysis of *ColLAC* genes

The coding sequences (CDS) of the 46 identified *LAC* genes from the *C. olitorius* genome-wide analysis were used as reference sequences for mapping short reads from jute tissue samples. Raw RNA-seq reads from leaves, roots, xylem (stem stick), and phloem (stem bast) tissues of *C. olitorius* were retrieved from the NCBI SRA database (BioProject PRJNA520880). For transcript quantification, the align_and_estimate_abundance.pl script was employed, and a gene expression matrix was generated using abundance_estimates_to_matrix.pl from the Trinity RNAseq suite (https://github.com/trinityrnaseq/trinityrnaseq; accessed on July 30, 2024). The read counts were TMM-normalised, log2-transformed, and visualised as a heatmap using MeV software (https://sourceforge.net/projects/mev-tm4/; accessed on July 30, 2024).

### 2.11 Identification of miRNA targets in *ColLAC*

The complete micro-RNA sequences of *A. thaliana* were acquired from PmiREN2.0 (Guo et al., 2022). The *ColLAC* sequences and the *Arabidopsis* miRNA sequences were subsequently submitted to psRNATarget to predict the target sites on the identified *ColLAC* genes (Dai and Zhao, 2011).

### 2.12 RNA isolation and cDNA synthesis

RNA was extracted from leaf, stem and root tissues using a commercially available RNA isolation kit (Macherey-Nagel, Germany). Following the manufacturer’s methodology, a tissue sample weighing 100 mg was pulverised into a fine powder using liquid nitrogen and a mortar-pestle. In order to eradicate any DNA contamination, a DNase treatment procedure was carried out in accordance with the instructions provided by the kit. The integrity of the RNA was evaluated by the utilisation of 2% agarose gel electrophoresis, while the RNA concentration was determined utilising a spectrophotometer (NanoDrop-2000c by ThermoScientific, USA). The synthesis of first-strand cDNA was performed using 1 μg of RNA and a first-strand cDNA synthesis kit (ThermoScientific, USA) according to the kit specified methodology.

### 2.13 PCR primers

The primers for this investigation were constructed using the PrimerQuest tool supplied by Integrated DNA Technologies (IDT), USA. The tool can be accessed at https://www.idtdna.com/PrimerQuest. The qRT-PCR primer design utilised default settings, which specified a melting temperature range of 59–62°C, a GC content range of 35–65%, a primer length range of 17–30 bp, and an amplicon size range of 75–150 bp. The comprehensive inventory of primers can be seen in **Supplementary Table 1**.

### 2.14 Gene expression analysis in qRT-PCR

The qRT-PCR analysis was conducted using the QuantStudio™ 5 machine (ThermoScientific, USA) which was equipped with QuantStudio™ Design and Analysis Software version 1.5.2. The recently produced complementary DNA (cDNA) was mixed with molecular-grade water in a 1:9 ratio before being utilised as a sample in qRT-PCR. The PCR cycle conditions used in the experiment were as follows: an initial denaturation step at 95°C for 2 minutes, followed by 40 cycles of denaturation at 95°C for 15 seconds, annealing at the temperature specific to the primer (as indicated in **Supplementary Table 1**) for 1 minute, and extension at 95°C for 15 seconds. The temperature range used to obtain the melt curve of the amplified PCR products was from 60°C to 95°C. The jute *ubiquitin1* (*UBI*) gene, with the GenBank number GH985256, used as an internal control (Parida et al., 2024b). The expression levels of the mRNA transcript were determined and depicted using relative quantification (RQ) values, following the approach outlined by Livak and Schmittgen (2001). Three technical replicates were conducted for each biological replication in the qRT-PCR study, with a total of three biological replicates.

### 2.15 *ColLAC*s gene expression analysis

Seven Arabidopsis genes implicated in the lignification process were selected based on prior research (Berthet et al., 2012). The genes include *AtLAC2* (AT2G29130), *AtLAC4* (AT2G38080), *AtLAC5* (AT2G40370), *AtLAC10* (AT5G01190), *AtLAC11* (AT5G03260), *AtLAC12* (AT5G05390), and *AtLAC17* (AT5G60020). The *AtLAC* genes were utilised as queries in a BLAST search to identify their homologous genes in the tossa jute genome using TBtools software (Chen et al., 2020). Genes categorised as *ColLAC*, demonstrating over 60% similarity with *AtLAC* sequences, were considered homologous and selected for subsequent expression analysis via qRT-PCR (Jha et al., 2024). We analysed the expression of homologous *ColLAC* genes in the phloem tissue of white jute plants at different developmental stages (30 DAS, 60 DAS, 90 DAS, and 120 DAS) to explore the lignification process.

Homologs of the *Arabidopsis* laccase genes involved in the lignin pathway—*ColLAC3*, *ColLAC22*, *ColLAC30*, *ColLAC32*, *ColLAC34*, *ColLAC38*, *ColLAC40*, *ColLAC42*, and *ColLAC46*—were also examined in the phloem tissue of the tossa jute mutant *bfs* and its control, JRO632, at different developmental stages up to 120 DAS.

### 2.16 Statistical analysis

The average Ct values were calculated using Microsoft Excel. For detailed statistical analysis, GraphPad Prism (version 9.0) software (https://www.graphpad.com/features) was used. A one-way analysis of variance (ANOVA, F-test) was performed to evaluate the significance of *ColLAC*s expression differences across different tissue samples (leaf, stem and root) and time points (30 DAS, 60 DAS, 90 DAS and 120 DAS), with a significance threshold of *P* ≤ 0.05. Prior to ANOVA, the data were tested for normality and homogeneity of variances to ensure compliance with statistical assumptions. Tukey’s *post hoc* test was then applied for multiple comparisons among the groups, specifically to compare *ColLACs* expression between JRO632 and *bfs*. Significant differences are indicated by asterisks, while non-significant values are denoted as ‘ns’. The expression data for *ColLACs* are presented as the mean of three biological replicates.

## 3 Results

### 3.1 Number of tossa jute laccase (*ColLAC*s)

**Figure 2:**
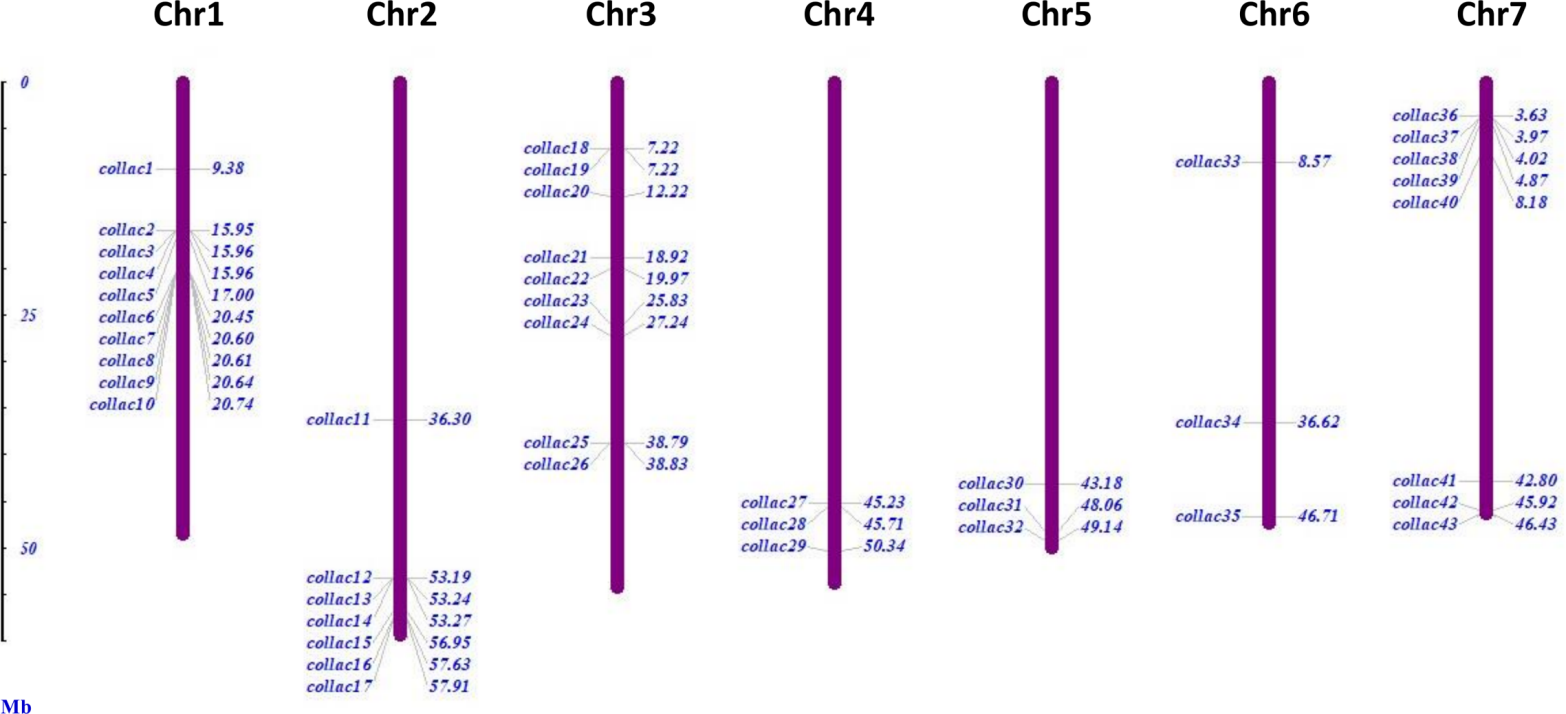
Chromosomal distribution of 43 laccase (*ColLAC*) genes in the genome of the tossa jute plant (*C. olitorius*). The remaining three genes, *ColLAC44* and *ColLAC45*, were mapped to contig 000000108, and *ColLAC46* was mapped to contig 000000440.

There were 46 laccase proteins found in the genome of tossa jute (*C. olitorius*), and they were called *ColLAC1* to *ColLAC46* according to their chromosomal locations (**Table 1**). The *LAC* genes that were discovered exhibited an inconsistent distribution throughout the seven chromosomes, as shown in **Figure 2**. Chromosome 1 has the most number of genes, namely 10 *LAC* genes. Chromosome 3 with 9 genes, chromosome 7 with 8 genes, chromosome 2 with 7 genes, and three *LAC* genes were detected on chromosomes 4, 5, and 6, respectively. Three *LAC* members *ColLAC44, ColLAC45*, and *ColLAC46* were located on the scaffold.

**Table 1:** Bioinformatics analysis and physicochemical properties of tossa jute laccases (*ColLACs*).

Gene Name	Gene ID	Gene Length (bp)	Position	Amino Acid (aa)	pI	MW (kDa)	Subcellular Localization
*ColLAC1*	GWHPBCLB000632	465	Chr1	119	4.92	13.36	Cytoplasm
*ColLAC2*	GWHPBCLB001017	388	Chr1	104	5.48	11.88	Cytoplasm
*ColLAC3*	GWHPBCLB001020	356	Chr1	102	5.14	11.27	Cytoplasm
*ColLAC4*	GWHPBCLB001021	645	Chr1	111	9.44	12.46	Cytoplasm
*ColLAC5*	GWHPBCLB001057	2426	Chr1	562	8.00	63.49	Peroxisome
*ColLAC6*	GWHPBCLB001225	2518	Chr1	560	5.40	61.94	Vacuoles
*ColLAC7*	GWHPBCLB001232	1248	Chr1	295	5.18	32.03	Vacuoles
*ColLAC8*	GWHPBCLB001233	14137	Chr1	1077	5.88	120.22	Chloroplast
*ColLAC9*	GWHPBCLB001234	2457	Chr1	561	5.60	62.42	Endoplasmic reticulum
*ColLAC10*	GWHPBCLB001236	2435	Chr1	525	5.37	58.16	Cytoskeletons
*ColLAC11*	GWHPBCLB004704	5046	Chr2	594	6.07	66.49	Vacuoles
*ColLAC12*	GWHPBCLB005880	2119	Chr2	579	8.59	65.31	Cytoplasm
*ColLAC13*	GWHPBCLB005882	2268	Chr2	588	6.34	65.80	Chloroplast
*ColLAC14*	GWHPBCLB005887	2515	Chr2	594	9.05	66.58	Cytoplasm
*ColLAC15*	GWHPBCLB006273	9237	Chr2	1074	9.46	120.09	Vacuoles
*ColLAC16*	GWHPBCLB006344	2343	Chr2	600	7.07	67.13	Cytoskeletons
*ColLAC17*	GWHPBCLB006382	5987	Chr2	856	8.91	95.24	Cytoplasm
*ColLAC18*	GWHPBCLB006781	224	Chr3	74	6.70	7.98	Cytoskeletons
*ColLAC19*	GWHPBCLB006782	772	Chr3	141	5.54	15.30	Extracellular space
*ColLAC20*	GWHPBCLB007074	2270	Chr3	572	5.62	63.71	Chloroplast
*ColLAC21*	GWHPBCLB007577	3147	Chr3	554	8.70	61.01	Chloroplast
*ColLAC22*	GWHPBCLB007668	2655	Chr3	576	8.53	63.75	Chloroplast
*ColLAC23*	GWHPBCLB008272	3478	Chr3	654	7.23	73.34	Nucleus
*ColLAC24*	GWHPBCLB008400	3010	Chr3	616	8.84	69.32	Vacuoles
*ColLAC25*	GWHPBCLB009266	1282	Chr3	340	6.80	37.99	Vacuoles
*ColLAC26*	GWHPBCLB009268	926	Chr3	186	8.56	20.71	Vacuoles
*ColLAC27*	GWHPBCLB012739	2606	Chr4	557	9.04	62.56	Vacuoles
*ColLAC28*	GWHPBCLB012804	3960	Chr4	542	9.22	60.28	Vacuoles
*ColLAC29*	GWHPBCLB013347	2167	Chr4	544	8.71	60.55	Chloroplast
*ColLAC30*	GWHPBCLB016154	2150	Chr5	562	9.25	62.64	Chloroplast
*ColLAC31*	GWHPBCLB016620	3009	Chr5	574	9.26	63.72	Chloroplast
*ColLAC32*	GWHPBCLB016731	2282	Chr5	546	9.28	59.48	Vacuoles
*ColLAC33*	GWHPBCLB017328	3358	Chr6	578	7.29	64.65	Chloroplast
*ColLAC34*	GWHPBCLB018816	2189	Chr6	566	8.83	62.38	Chloroplast
*ColLAC35*	GWHPBCLB019837	3451	Chr6	542	9.57	60.93	Cytoplasm
*ColLAC36*	GWHPBCLB020385	1755	Chr7	306	6.05	34.36	Cytoplasm
*ColLAC37*	GWHPBCLB020416	2612	Chr7	583	9.31	64.30	Chloroplast
*ColLAC38*	GWHPBCLB020423	2415	Chr7	581	9.24	63.87	Chloroplast
*ColLAC39*	GWHPBCLB020530	2209	Chr7	575	9.48	63.50	Chloroplast
*ColLAC40*	GWHPBCLB020902	3035	Chr7	560	9.16	61.31	Chloroplast
*ColLAC41*	GWHPBCLB022673	8853	Chr7	956	8.40	107.22	Plastid
*ColLAC42*	GWHPBCLB023001	13783	Chr7	1548	6.62	173.99	Cytoplasm
*ColLAC43*	GWHPBCLB023076	2691	Chr7	571	7.24	63.50	Cytoplasm
*ColLAC44*	GWHPBCLB023256	12749	tig00000108	1078	8.15	121.05	Chloroplast
*ColLAC45*	GWHPBCLB023259	2554	tig00000108	568	7.26	64.35	Cytoplasm
*ColLAC46*	GWHPBCLB023867	2165	tig00000440	559	8.30	61.74	Extracellular space

### 3.2 Laccase proteins

The length of these ColLAC proteins varied, with the smallest protein (ColLAC18) having a length of 7.98 kDa and the largest protein (ColLAC42) having a length of 173.99 kDa. The ColLAC proteins exhibited a maximum of 1548 amino acids, a minimum of 74 amino acids, and an average of around 554 amino acids (**Table 1**). The instability index of the discovered LAC proteins was projected to be less than 40, except for ColLAC1 (45.18), ColLAC2 (41.63), ColLAC4 (52.92), ColLAC19 (41.84), ColLAC42 (41.14), and ColLAC43 (43.48) (**Supplementary Table 2**). This suggests that most of the proteins are stable. Furthermore, the aliphatic amino acid index of ColLAC1 was the lowest at 72.02, whereas ColLAC4 had the highest value at 111.35. The overall mean hydropathy (GRAVY) score for all the *ColLACs* was negative (<0), demonstrating their hydrophilic characteristic (Kyte and Doolittle, 1982). The ColLAC proteins that were discovered were predicted to be located in different organelles of white jute, as shown in **Supplementary Table 2**. Our analysis revealed that 48.83% (21 out of 43) of ColLACs were identified as transmembrane proteins (**Supplementary Figure 1**).

### 3.3 Motif of laccase proteins

**Figure 3:**
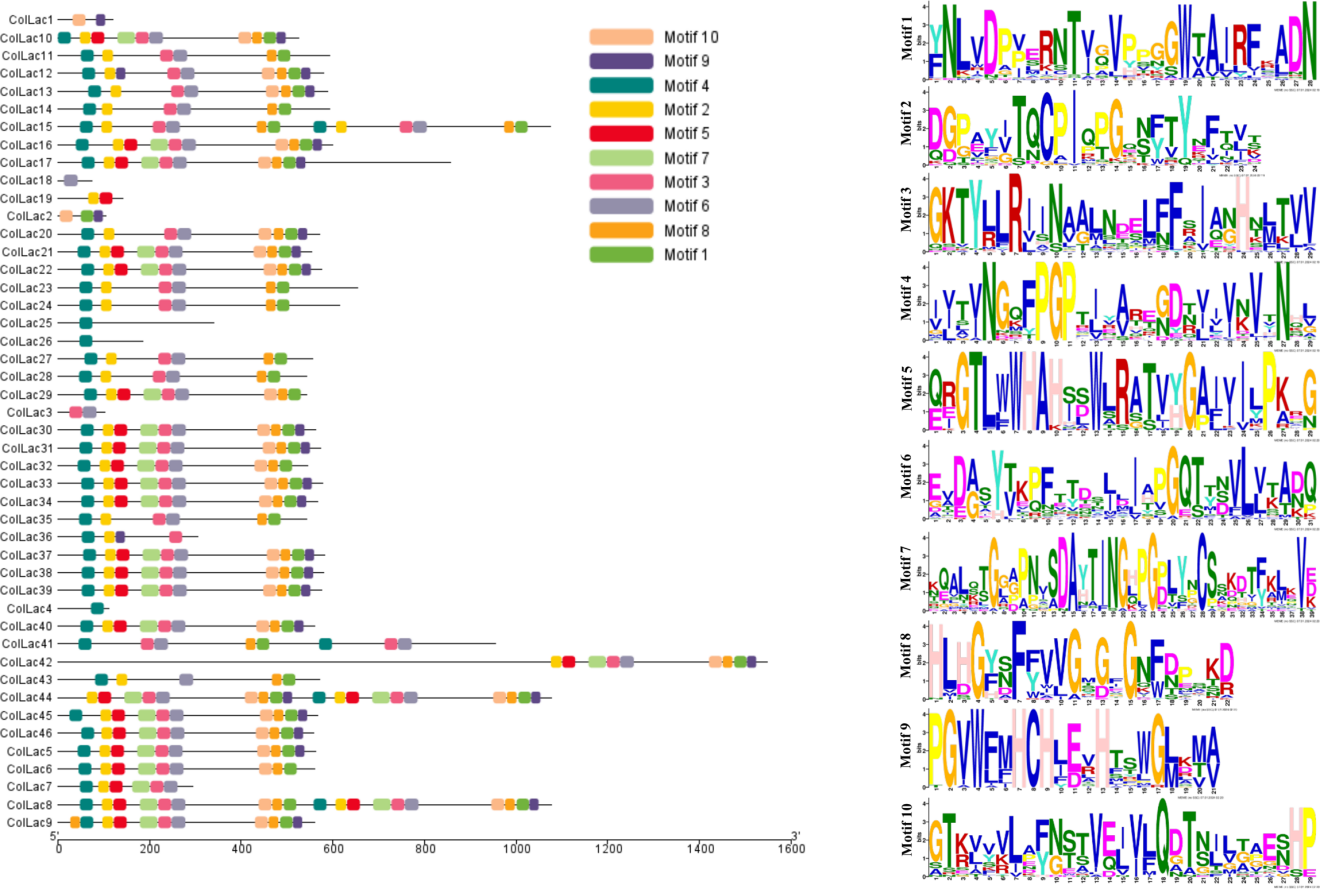
Distribution of conserved motifs in identified *ColLAC* proteins. Motifs are represented by distinct hues and their corresponding sequences.

The found laccase proteins were anticipated to include 10 patterns, labelled motif-1 through motif-10 (**Figure 3**). ColLAC4, ColLAC18, ColLAC25, and ColLAC26 exhibited single motifs, but ColLAC1, ColLAC3, and ColLAC19 had double motifs. Moreover, the laccase proteins ColLAC8, ColLAC15, and ColLAC44 exhibited repetitions of certain patterns.

### 3.4 Gene structure of *ColLACs*

**Figure 4:**
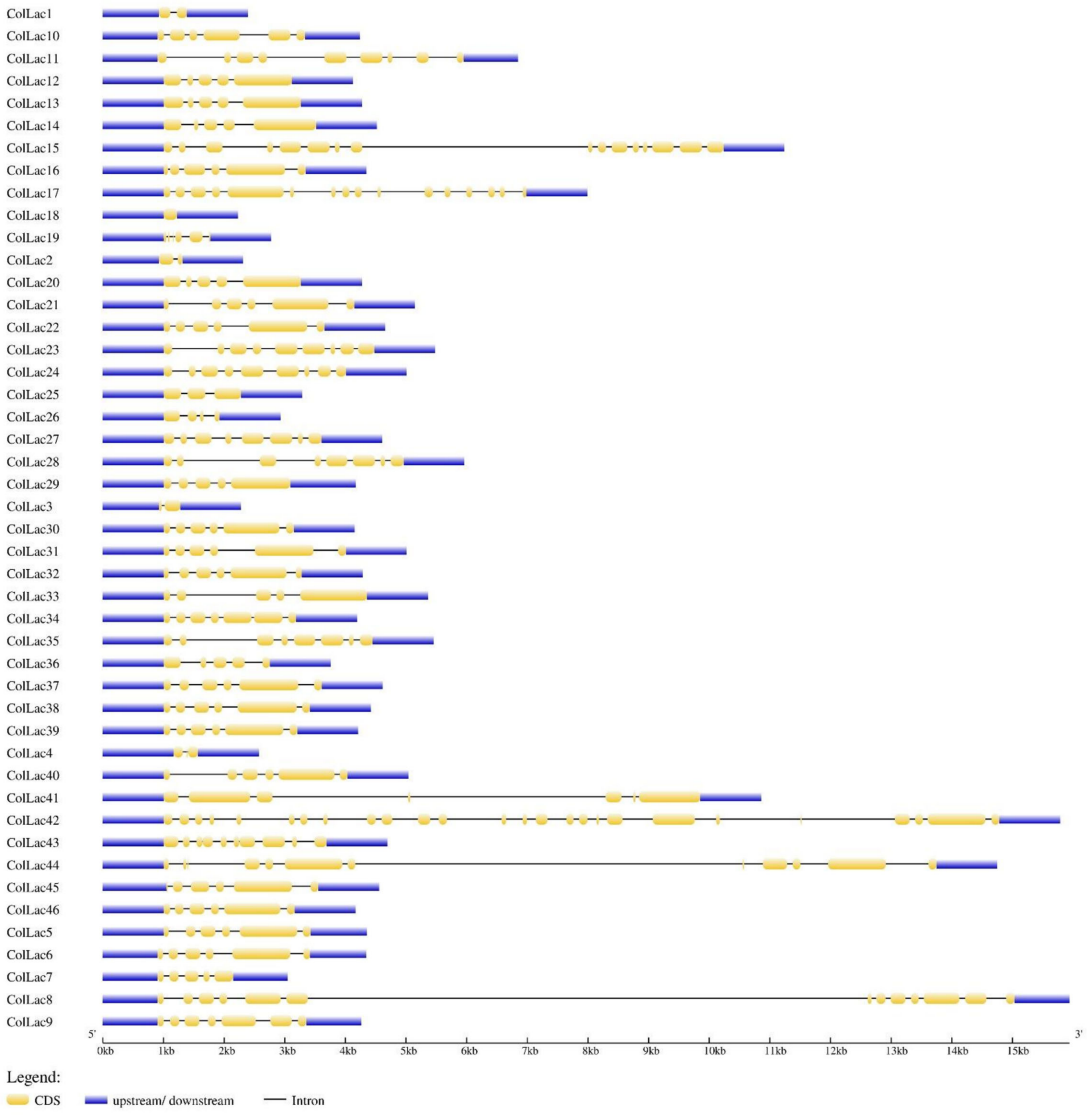
Gene architecture of 46 white jute laccase genes (*ColLAC*s).

The found *ColLAC* genes exhibited significant variability in exon-intron distribution, with exon counts ranging from 2 to 26. Only one exon was identified in the *ColLAC18* gene, while the *ColLAC42* gene had the greatest prediction of 26 exons (**Figure 4**).

### 3.5 Phylogeny of *ColLACs* proteins

**Figure 5:**
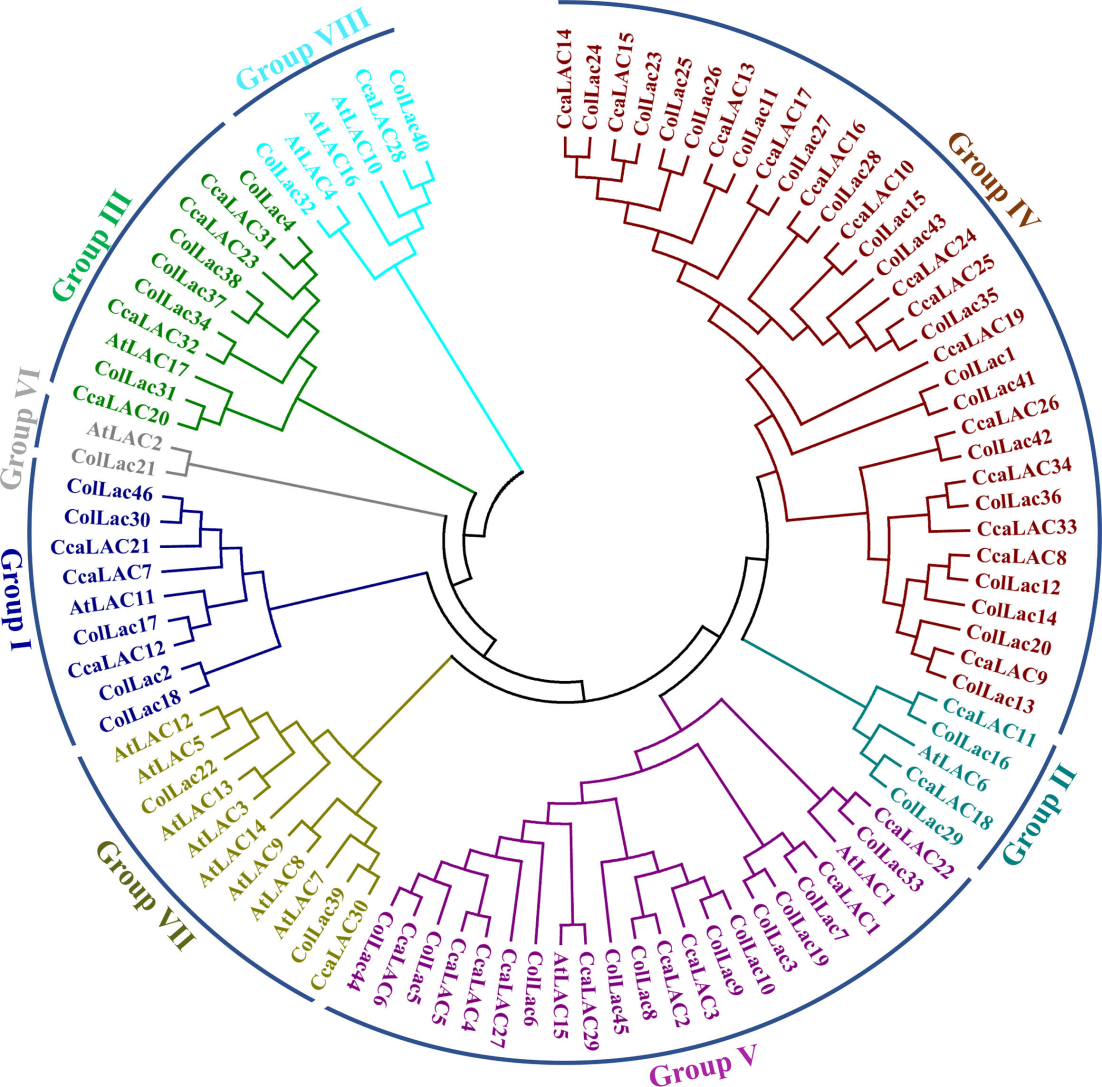
Phylogenetic tree of jute laccase proteins alongside laccase proteins from Arabidopsis (*A. thaliana*), white jute (*C. capsularis*), and tossa jute (*C. olitorius*). The phylogeny categorised the detected LAC members into eight categories.

The phylogenetic analysis divided the identified laccase proteins of tossa jute into eight groups, designated as Group I to Group VIII (**Figure 5**). The majority of laccase proteins were clustered in Group IV, which contained 18 members. Other groups, such as Group V, included 11 laccase proteins, while Group I and Group III each had 5 members. Groups II, VII, and VIII each contained 2 members, and only 1 member was found in Group VI.

### 3.6 Duplication of *ColLAC* genes

**Figure 6:**
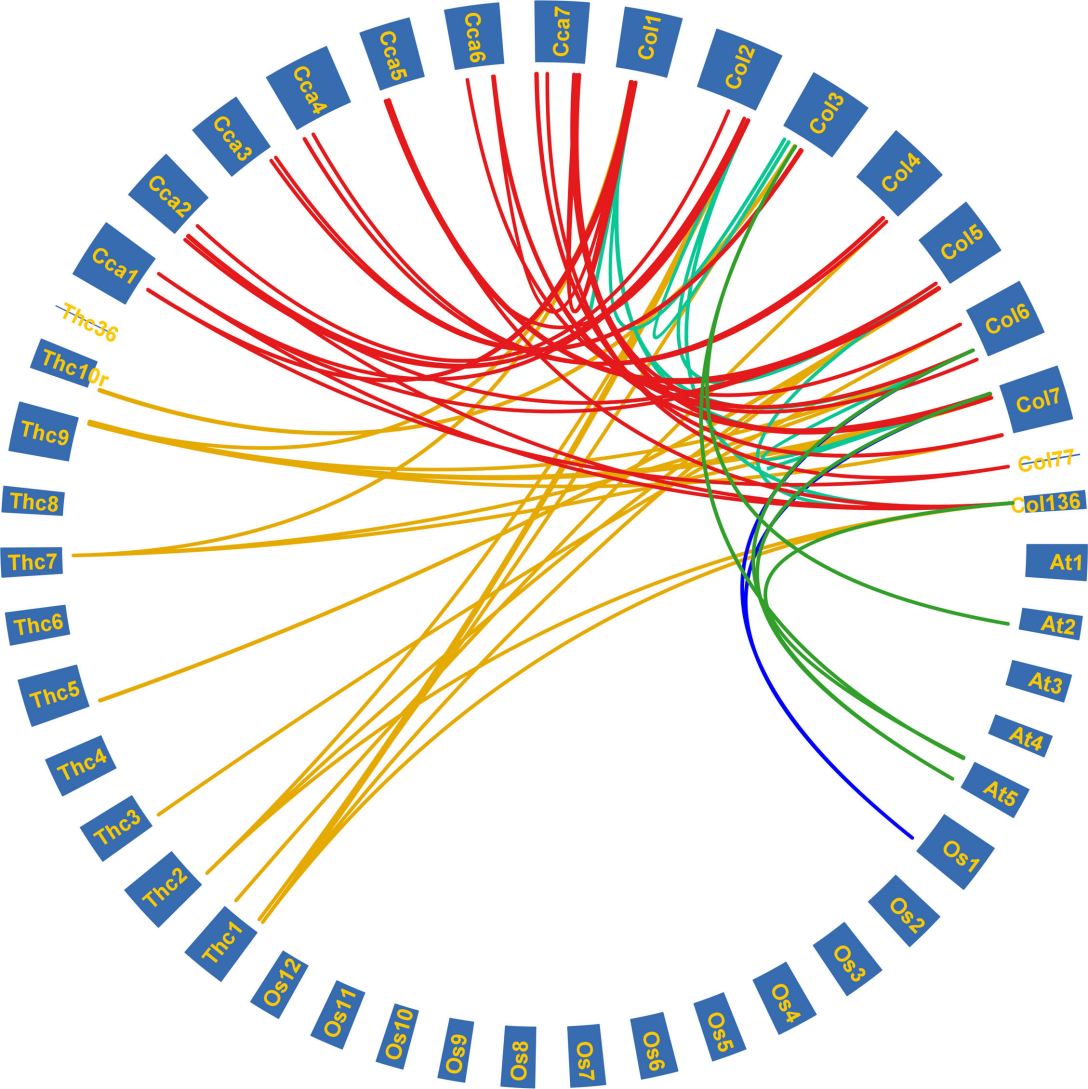
Syntenic relationships among the *LAC* genes of tossa jute (*C. olitorius*), Arabidopsis (*A. thaliana*), rice (*O. sativa*), cacao beans (*T. cacao*), and white jute (*C. capsularis*). Each coloured line signifies duplicated gene pairs across the species.

Gene duplication analysis revealed a total of 13 paralogous pairs, including 2 tandem duplications and 11 segmental duplications (**Supplementary Table 3**). Orthologous pairs were also identified: 5 pairs between *C. capsularis* and *A. thaliana*, 3 between *C. capsularis* and *O. sativa*, 26 between *C. capsularis* and *T. cacao*, and 55 pairs between *C. olitorius* and *C. capsularis* (**Figure 6**). Among species comparisons, the largest number of *LAC* orthologous pairs was found between *C. olitorius* and *T. cacao*, likely due to their close genomic relationship, as suggested by Sarkar et al. (2017).

### 3.7 Cis-acting elements

**Figure 7:**
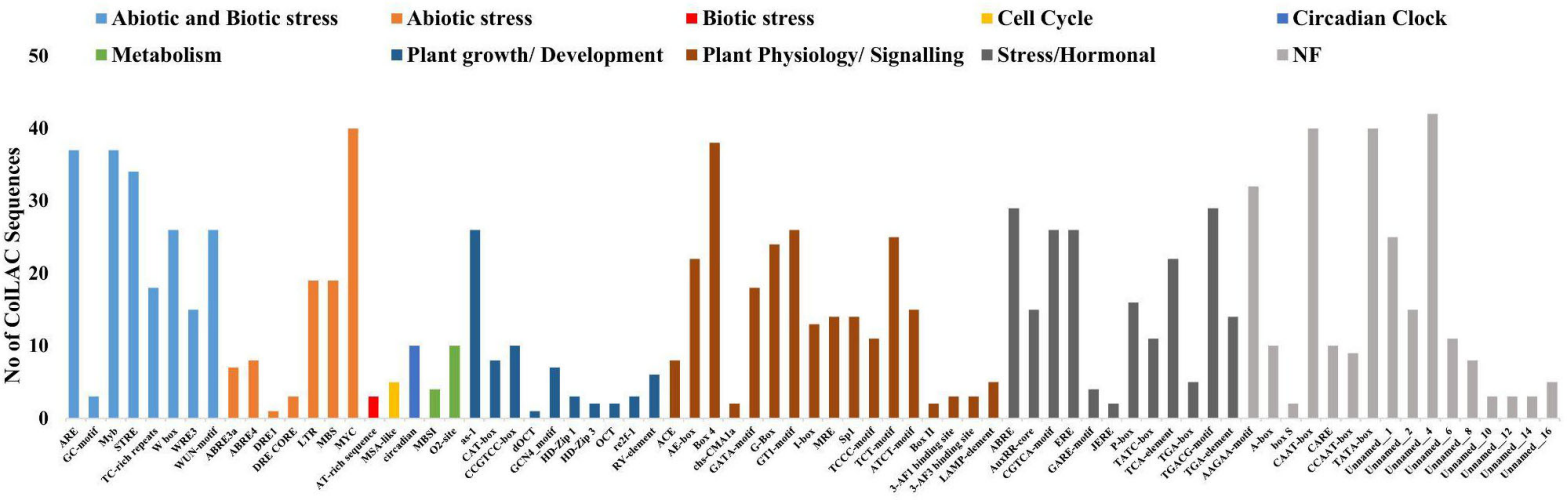
Cis-acting elements in the upstream regions of *ColLAC*s.

The upstream regions of *ColLAC* genes were found to contain various predicted cis-elements (**Figure 7**). Among the 46 *ColLAC* members, MYB, MYC, and TATA-box elements were consistently identified. Additionally, a variety of cis-elements involved in different biological processes were detected (Ain-Ali et al., 2021; Jha et al., 2024). These included elements linked to stress responses (such as STRE, W-box, MYB, MYC, and WUN-motif), hormonal signalling (including ABRE, ERE, GARE-motif, P-box, and TGA element), and developmental regulation (such as G-box, GT1, RY-element, Sp1, GATA-motif, LAMP element, Box-4, and TCT-motif). These results suggest that the promoter regions of *ColLAC* genes may contribute to multiple biological functions.

Upstream regions of multiple *ColLAC* genes contain cis-acting elements associated with secondary wall formation (**Supplementary Table 4**). These include AC elements (ACE), G-box motifs, MYB Binding Sites (MBS), and other regulatory elements involved in hormonal signalling and plant cell wall development (Zhou et al., 2009; Zhao et al., 2010; Sun et al., 2022).

### 3.8 Functional role analysis of *ColLAC* genes through gene ontology (GO) term assignment

GO term assignment revealed that the *ColLAC* genes are associated with several biological processes, including the phenylpropanoid metabolic process (GO:0009698), phenylpropanoid biosynthetic process (GO:0009699), lignin metabolic process (GO:0009808), lignin biosynthetic process (GO:0009809), and secondary metabolite process (GO:0019748) (**Supplementary Figure 2**). Furthermore, the GO analysis indicated that *ColLAC* genes are involved in various molecular functions, such as oxidoreductase activity (GO:0016491), copper ion binding (GO:0005507), transition metal ion binding (GO:0046914), ion binding (GO:0043167), metal ion binding (GO:0046872) and catalytic activity (GO:0003824) (**Supplementary Figure 2**). These molecular functions are consistent with those typically associated with the laccase gene family.

### 3.9 Tissue-specific expression of *ColLAC* genes

**Figure 8:**
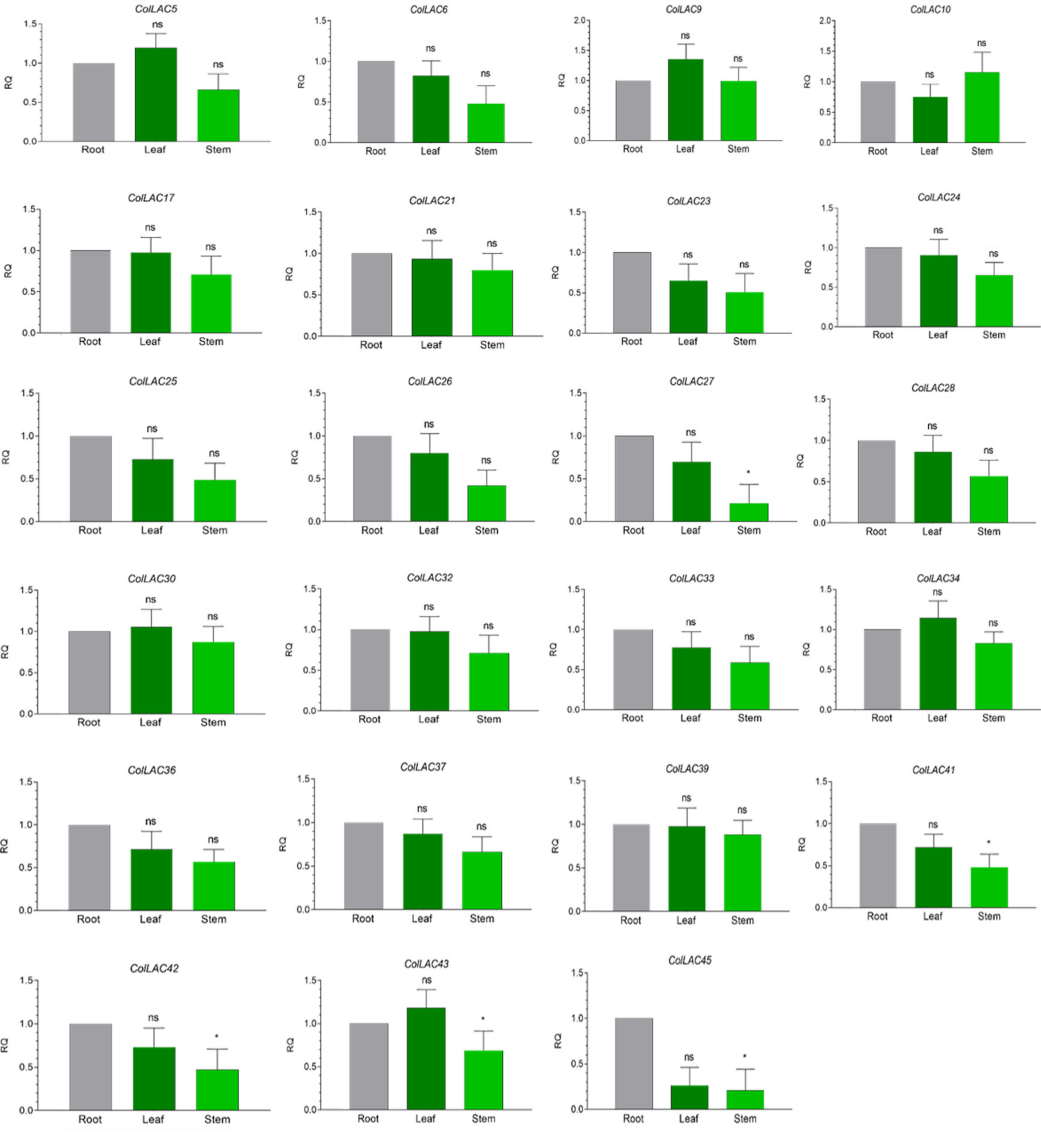
Tissue-specific consistent expression of laccase (*ColLAC*) genes. This figure illustrates the relative quantification (RQ) values of *ColLAC* gene expression in the leaf, stem, and root tissues of tossa jute. Asterisks indicate significant differences in *P* values from Tukey’s test, whereas non-significant values are labelled as ‘ns’.

**Figure 9:**
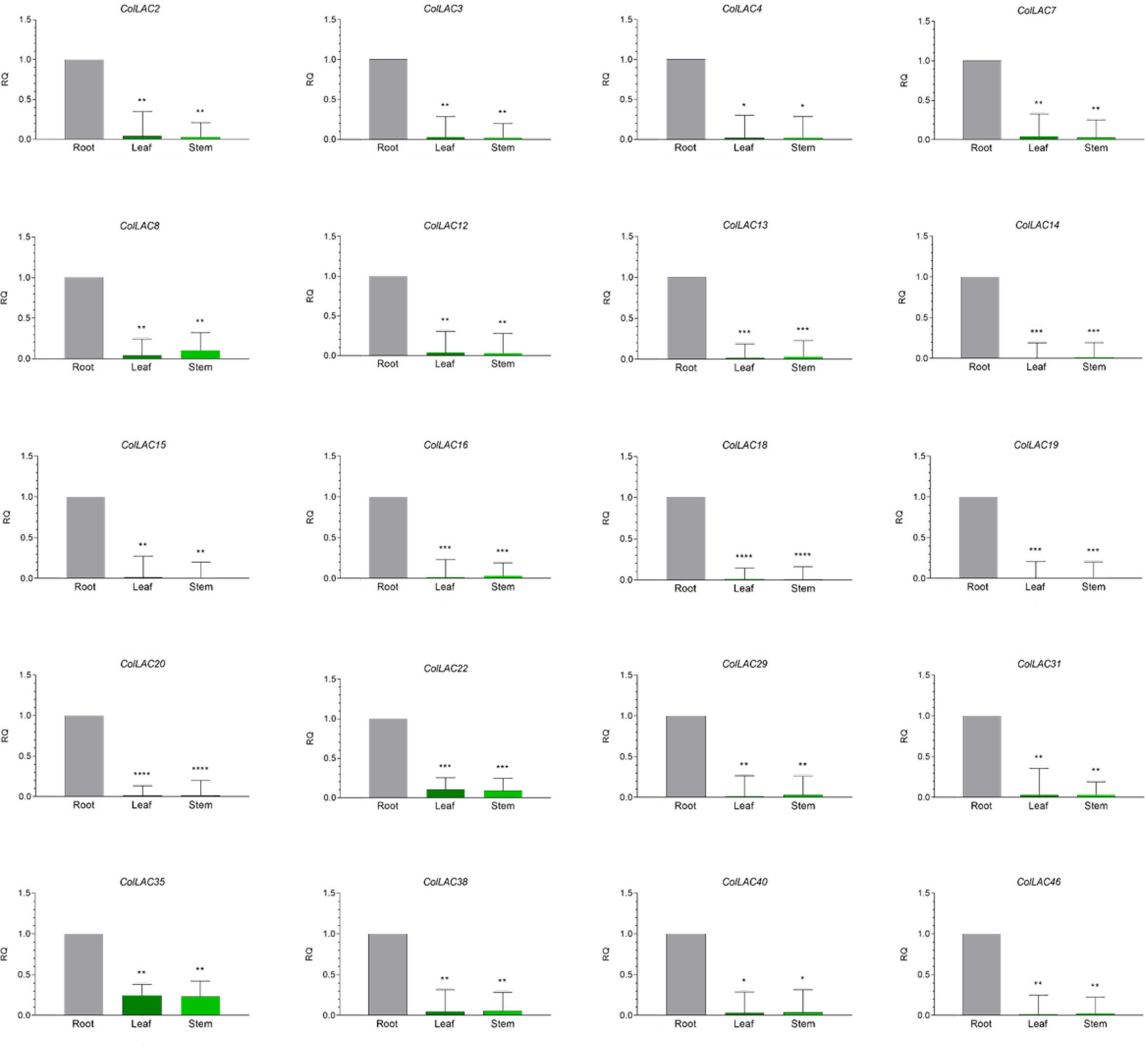
Tissue-specific "variable" expression of laccase (*ColLAC*) genes. This figure shows the relative quantification (RQ) values of *ColLAC* gene expression in the leaf, stem, and root tissues of tossa jute. Asterisks denote significant differences in *P* values from Tukey’s test.

The expression levels of all 46 *ColLAC* genes across three different tissues (leaf, stem, and root) were analysed using qRT-PCR. The mRNA transcript levels were presented as relative quantification (RQ) values, with root tissue serving as the reference point (set to an RQ value of 1). Two distinct expression patterns were identified: one group of *ColLAC* genes exhibited consistent expression across the leaf, stem, and root tissues (**Figure 8**), while another group showed significantly higher expression in root tissue compared to the leaf and stem (**Figure 9**).

Consistent expression of *ColLAC* genes was observed in *ColLAC5*, *ColLAC6*, *ColLAC9*, *ColLAC10*, *ColLAC17*, *ColLAC21*, *ColLAC23*, *ColLAC24*, *ColLAC25*, *ColLAC26*, *ColLAC27*, *ColLAC28*, *ColLAC30*, *ColLAC32*, *ColLAC33*, *ColLAC34*, *ColLAC36*, *ColLAC37*, *ColLAC39*, *ColLAC41*, *ColLAC42*, *ColLAC43*, and *ColLAC45*.

Significantly higher expression in root tissue compared to leaf and stem was observed in *ColLAC2*, *ColLAC3*, *ColLAC4*, *ColLAC7*, *ColLAC8*, *ColLAC12*, *ColLAC13*, *ColLAC14*, *ColLAC15*, *ColLAC16*, *ColLAC18*, *ColLAC19*, *ColLAC20*, *ColLAC22*, *ColLAC29*, *ColLAC31*, *ColLAC35*, *ColLAC38*, *ColLAC40*, and *ColLAC46*.

### 3.10 *In-silico* expression analysis of *ColLACs*

**Figure 10:**
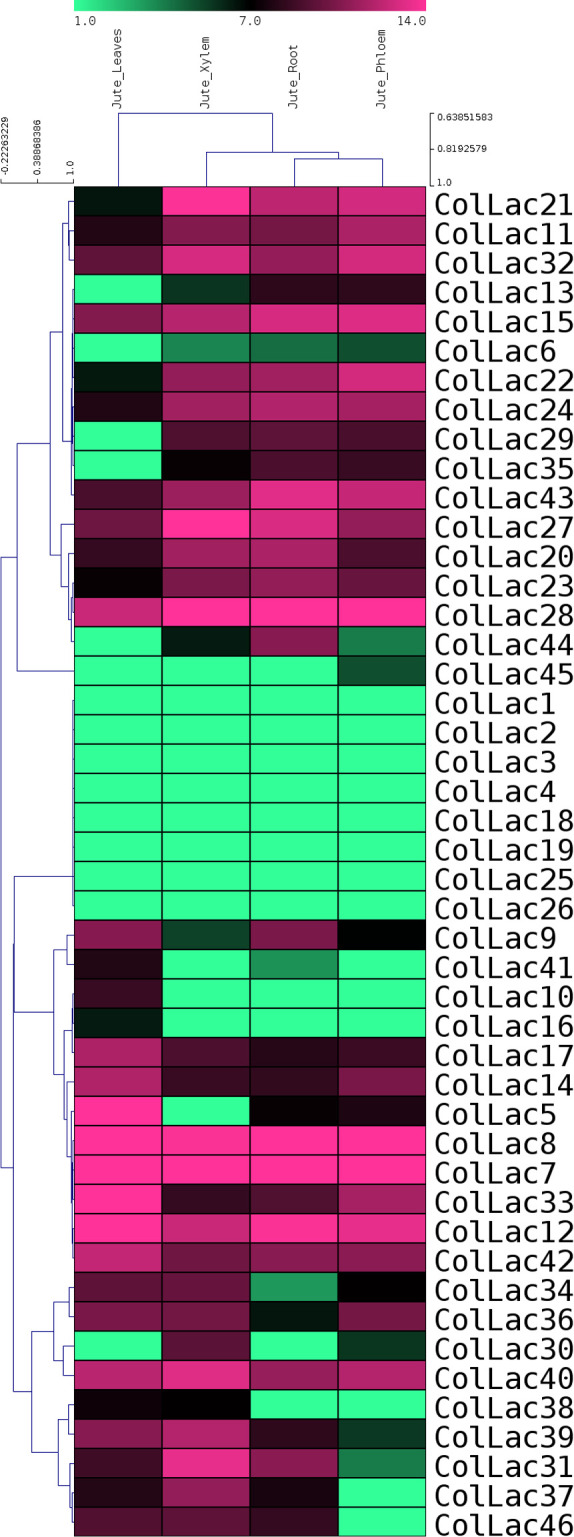
*In-silico* expression study of laccase genes in tossa jute (*ColLAC*s) across leaf, phloem, xylem, and root tissues. Heat map derived from publicly accessible transcriptomics data from BioProject PRJNA520880.

Publicly available transcriptomic data from BioProject PRJNA520880 on various tissues of tossa jute were used for *in-silico* expression analysis of *ColLAC* genes. The expression levels of all 46 *ColLAC* genes in leaf, phloem, xylem, and root tissues were analysed and visualised through a heatmap (**Figure 10**). Nine *ColLAC* genes were randomly selected from the heatmap for validation of their expression via qRT-PCR. The qRT-PCR results showed a similar expression pattern to that observed in the transcriptomic heatmap (**Supplementary Figure 3**). In the heatmap, most *ColLAC* genes exhibited higher expression in root tissue compared to leaf, phloem, and xylem.

### 3.11 *ColLAC* gene expression at different developmental stages

Nine genes from the lignin biosynthesis pathway in *Arabidopsis* were selected, and their homologous counterparts in jute, *ColLAC3*, *ColLAC22*, *ColLAC30*, *ColLAC32*, *ColLAC34*, *ColLAC38*, *ColLAC40*, *ColLAC42*, and *ColLAC46*, were identified for expression analysis at different developmental stages (30 DAS, 60 DAS, 90 DAS, and 120 DAS) in two tossa jute cultivars—JRO632 (control) and *bfs* (mutant). The expression of these genes was analysed in the phloem tissue, as jute produces phloem fibres, and the findings could provide insights into the lignification process of the fibres.

**Figure 11:**
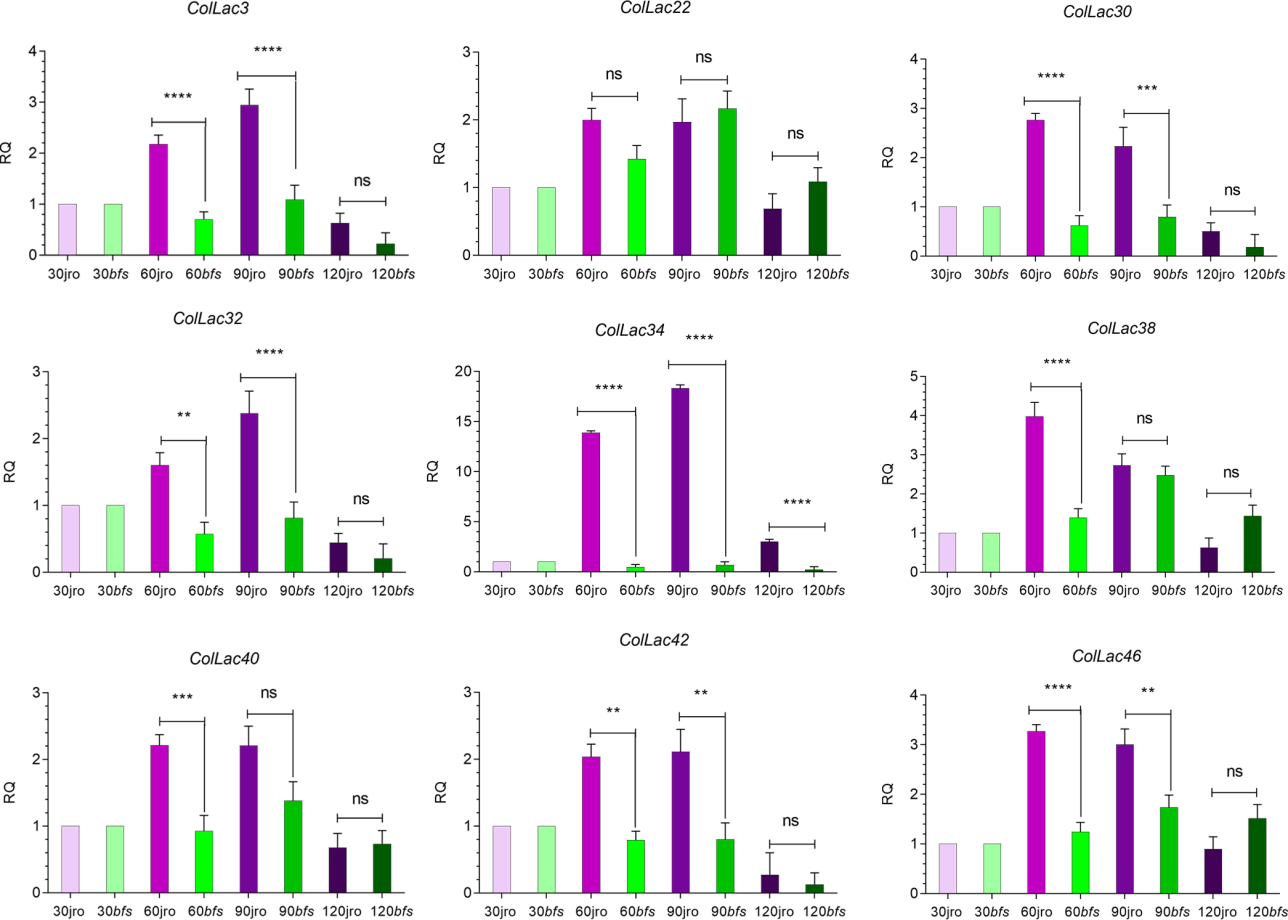
Expression profiles of *ColLAC* genes at different developmental stages (30, 60, 90, and 120 days after sowing, DAS) in tossa jute cultivars JRO632 (control) and *bfs* (mutant). Significant differences (*P* ≤ 0.05) are marked by asterisks, while non-significant values are labelled as ‘ns’.

In both cultivars, JRO632 and *bfs*, the highest expression of *ColLAC* genes was observed at 90 DAS. Significant differences in *ColLAC* expression between JRO632 and *bfs* were detected at 60 DAS and 90 DAS, while differences at 120 DAS were not statistically significant (**Figure 11**). Among all the genes, *ColLAC34* exhibited the most significant expression differences at all time points between the two cultivars. The expression pattern of *ColLAC34* suggests it may play a role in these developmental processes, as it exhibited significantly lower expression in the *bfs* mutant. However, further studies are required to fully understand its function in tossa jute.

### 3.12 Expression of *ColLAC* under copper stress

**Figure 12:**
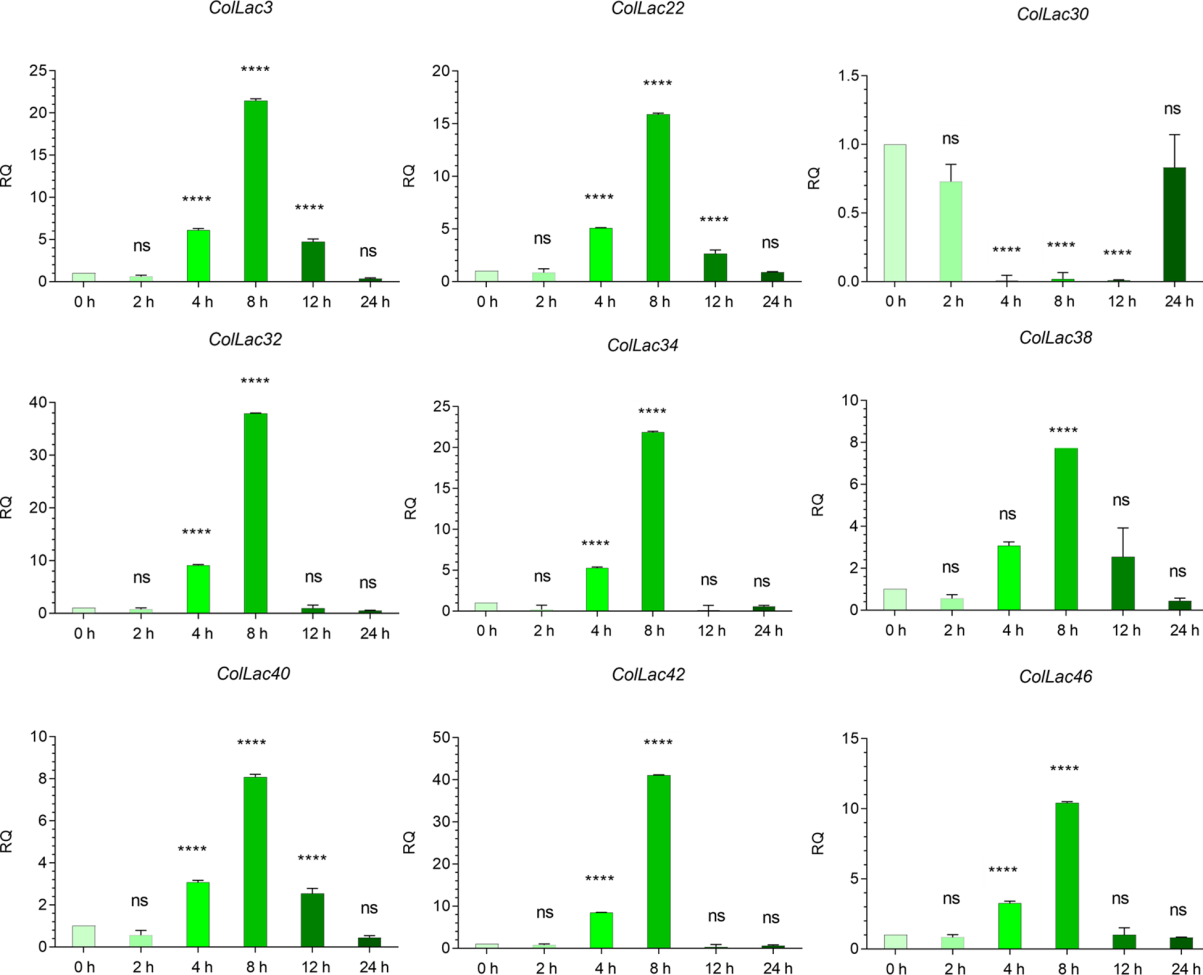
Expression profiles of *ColLAC* genes under copper stress (0.10 mM CuSO_4_·H_2_O treatment). Tukey’s multiple comparison test. Significant differences (*P* ≤ 0.05) are indicated by asterisks, while non-significant results are labelled as ‘ns’.

The expression of *ColLAC3*, *ColLAC22*, *ColLAC30*, *ColLAC32*, *ColLAC34*, *ColLAC38*, *ColLAC40*, *ColLAC42*, and *ColLAC46*—homologs of *Arabidopsis laccase* (*AtLAC*) genes involved in the lignin pathway—was examined in plants subjected to copper heavy metal stress (using 0.10 mM CuSO_4_·H_2_O). With the exception of *ColLAC30*, all other *ColLAC* genes showed peak expression at 8 hours of copper treatment (**Figure 12**). Although *ColLAC30* exhibited a minimal fold change compared to the other *ColLAC* genes, none of the tested genes showed significant expression differences at 24 hours compared to the starting point (0-hour samples). Furthermore, aside from *ColLAC3*, *ColLAC22*, and *ColLAC40*, no significant expression was observed at the 12-hour mark. Our findings suggest that *ColLAC* genes respond predominantly within the first 12 hours of copper stress, with the highest expression levels occurring at 8 hours.

### 3.13 Expression of *ColLAC* under ABA hormonal stress

**Figure 13:**
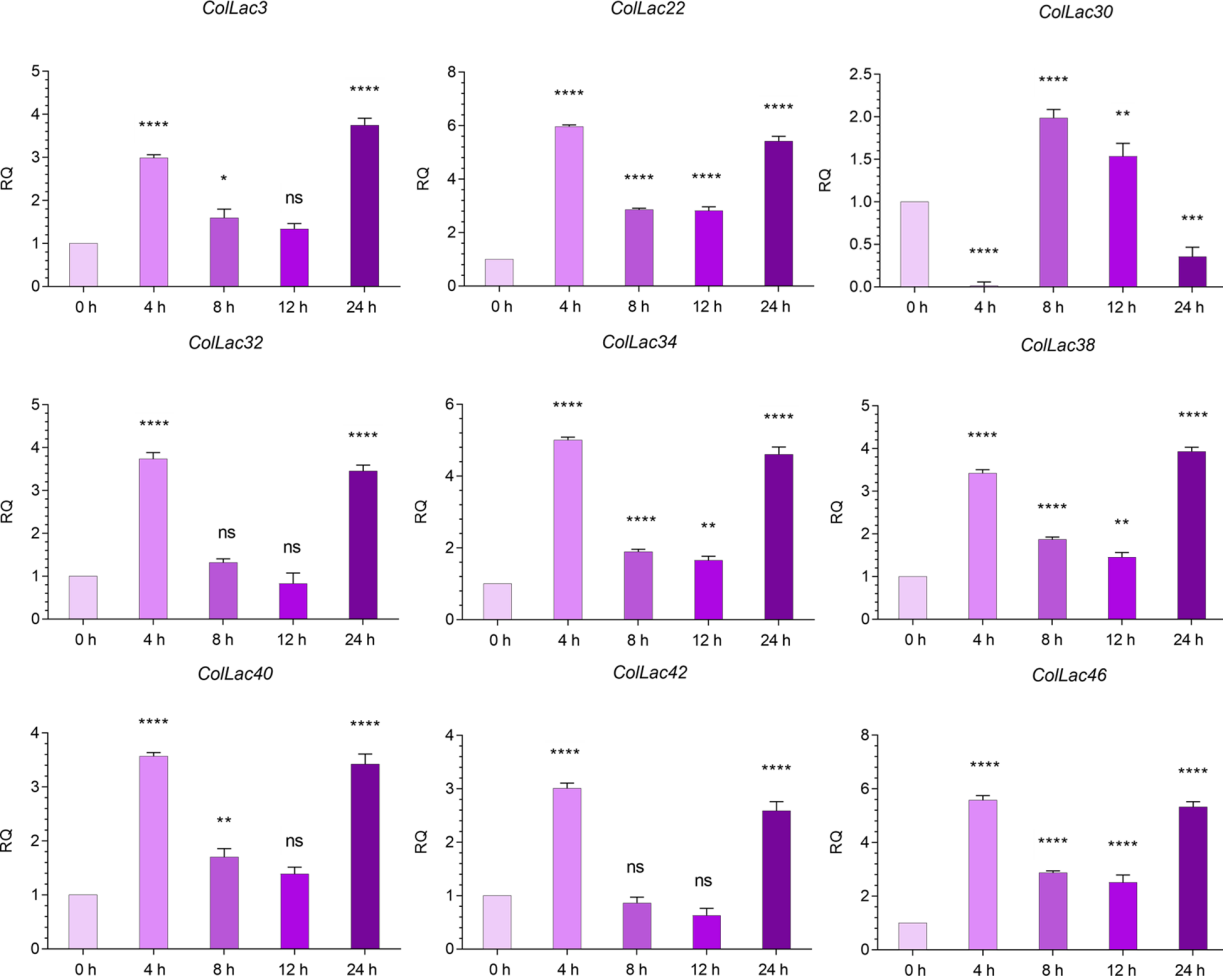
Expression profiles of *ColLAC* genes under ABA hormonal stress (using 0.15 mM ABA treatment). Significant differences (*P* ≤ 0.05) are indicated by asterisks, while non-significant results are labelled as ‘ns’.

The expression levels of *AtLAC*-homologous *ColLAC* genes, including *ColLAC3*, *ColLAC22*, *ColLAC30*, *ColLAC32*, *ColLAC34*, *ColLAC38*, *ColLAC40*, *ColLAC42*, and *ColLAC46*, were analysed under ABA treatment. With the exception of *ColLAC30*, a significant increase in gene expression was observed at 4 hours, reaching its peak at this time point (**Figure 13**). For *ColLAC30*, the highest expression was recorded at 8 hours. With the exception of *ColLAC30*, all the *ColLAC* genes exhibited a U-shaped expression pattern, with elevated levels at 4 and 24 hours, and reduced expression at 8 and 12 hours. In contrast, *ColLAC30* showed a gradual decline in expression from 8 to 24 hours.

### 3.14 *ColLAC* targeted by miRNA

**Figure 14:**
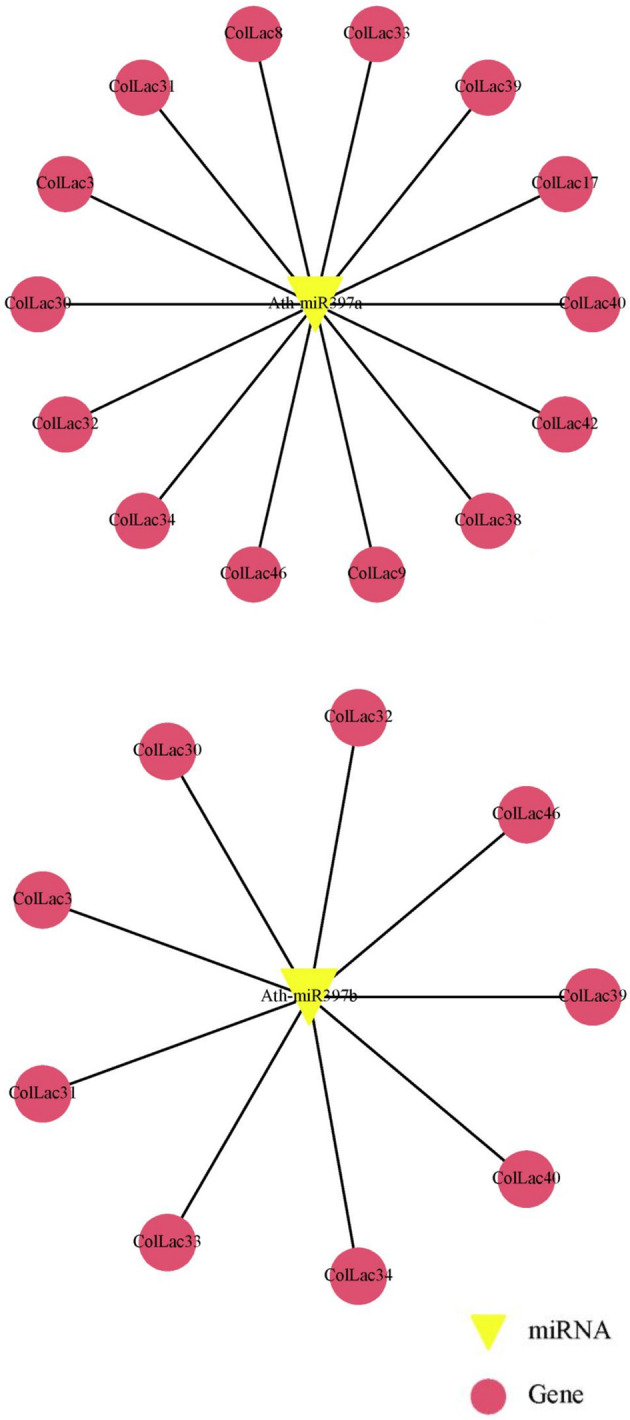
Prediction of miR397 target sites in *ColLAC* genes. Target sites for Arabidopsis miR397 (Ath-miR397a and Ath-miR397b) identified across multiple *ColLAC* genes.

The *laccase* genes in tossa jute (*ColLACs*) were identified as potential target sites for lignin-related miRNAs, particularly miR397 (**Supplementary Table 5**). Specifically, *Ath-miR397a* was predicted to target 14 *ColLAC* genes, including *ColLAC3*, *ColLAC8*, *ColLAC9*, *ColLAC17*, *ColLAC30*, *ColLAC31*, *ColLAC32*, *ColLAC33*, *ColLAC34*, *ColLAC38*, *ColLAC39*, *ColLAC40*, *ColLAC42*, and *ColLAC46*. In comparison, *Ath-miR397b* was predicted to target 9 *ColLAC* genes: *ColLAC3*, *ColLAC30*, *ColLAC31*, *ColLAC32*, *ColLAC33*, *ColLAC34*, *ColLAC39*, *ColLAC40*, and *ColLAC46* (**Figure 14**). Among these, eight genes—*ColLAC3*, *ColLAC30*, *ColLAC31*, *ColLAC32*, *ColLAC33*, *ColLAC34*, *ColLAC40*, and *ColLAC46*—were commonly targeted by both *Ath-miR397a* and *Ath-miR397b*.

## 4 Discussion

Studies on different plant laccases and their functionality are definitely not scarce. In *Arabidopsis*, mutations in *AtLAC4* and *AtLAC17* individually reduced lignin content by 10%, while double mutations in both genes (*AtLAC4* and *AtLAC17*) led to a 39% reduction (Berthet et al., 2011). *TaLAC4* silencing resulted in decreased lignin deposition in wheat (*Triticum aestivum*) stems (Soni et al., 2020). Overexpression of *PbLAC1* increased lignin content and promoted cell wall growth in pear (*Pyrus bretschneideri*) (Cheng et al., 2019). Similarly, *MsLAC1* overexpression resulted in higher lignin content in silvergrass (*Miscanthus* sp.) (He et al., 2019). Downregulation of *GhLAC1* led to increased fibre initials and reduced fibre lengths in cotton (*Gossypium herbaceum*) (Hu et al., 2020). Functional studies on jute *LAC* genes were lacking until recently, when we reported the presence of 34 *C. capsularis* laccase (*CcaLACs*) genes (Parida et al., 2024a).

In white jute (*C. capsularis*), 11.76% of the *CcaLAC* genes (4 out of 34) were predominantly expressed in the roots. However, in tossa jute (*C. olitorius*), we observed a significantly higher percentage, with 43.47% of the *ColLAC* genes (20 out of 46) showing predominant expression in the roots (**Figure 9**). Additionally, 50% of the *ColLAC* genes (23 out of 46) exhibited no specific tissue preference (**Figure 8**), while 6.52% (3 out of 46) had undetectable expression in the tested tissues—leaves, stems, and roots. The high root-specific expression of *ColLAC* genes, compared to the *CcaLAC* genes, caught our attention.

To ensure accuracy, we repeated the tissue-specific experiments, validating the expression of *ColLAC* genes in two tossa jute cultivars, JRO524 and JRO632, and consistently found similar results. Similar root-specific expression patterns have been observed in other species, such as *Eucalyptus* laccases (*EgrLAC*s), where 58% of the *EgrLAC* genes showed root predominance (Arcuri et al., 2020). Rice laccases (*OsLAC*s) also demonstrated high expression in root tissues during vegetative stage (Liu et al., 2017), aligning with our findings in tossa jute *ColLAC*s. These results suggest that *LAC* gene expression in jute is species-specific, with significant differences between them. The detailed functionality study of laccases in roots is yet to be explored, but their presence strongly suggests a contribution to lignification.

To identify the *LAC* genes involved in tossa jute lignification, we selected a few homologous genes well studied as key players in Arabidopsis lignification. The rationale for this approach is based on the fact that Arabidopsis is an ideal model for plant lignification studies (Vanholme et al., 2010). There is extensive research available on Arabidopsis *LAC* genes (*AtLAC2, AtLAC4, AtLAC5, AtLAC10, AtLAC11, AtLAC12*, and *AtLAC17*) confirming their involvement in lignin biosynthesis (Berthet et al., 2011). In this study the protein sequences of Arabidopsis *LAC* genes were compared with those of tossa jute; after which the two most similar jute genes were selected based on sequence homology for further analysis across different developmental stages.

The sequence similarity analysis revealed that *AtLAC2* shared homology with *ColLAC3* (76.74%) and *ColLAC38* (74.32%), *AtLAC4* with *ColLAC40* (78.41%) and *ColLAC32* (75.81%), *AtLAC5* with *ColLAC22* (80.62%) and *ColLAC42* (66.26%), *AtLAC10* with *ColLAC32* (73.82%) and *ColLAC40* (73.56%), *AtLAC11* with *ColLAC46* (80.10%) and *ColLAC30* (79.85%), *AtLAC12* with *ColLAC22* (80.85%) and *ColLAC42* (65.91%), *AtLAC15* with *ColLAC1* (63.63%), and *AtLAC17* with *ColLAC34* (80.27%) and *ColLAC38* (80.13%).

New insights about gene expression patterns were gained by studying these homologous genes at different development stages of the phloem tissue. Gene expression showed a steady rise from 30 DAS (when lignification begins) to 120 DAS (when the plants are harvested for lignocellulosic fibres). This steady rise suggests the contribution of *LAC* genes in the lignification process and structural development. To further investigate their potential role in structural development, we utilised the X-ray-irradiated mutant *bfs* (bast fibre-shy), which exhibits structural abnormalities (Kundu et al., 2012). Consequently, the *ColLAC34* gene emerged as a candidate gene that could potentially have a dual role in lignification and structural buildout in tossa jute as it is homologous to *AtLAC17*. The *AtLAC17* is a well-characterised lignification gene in Arabidopsis (Berthet et al., 2011). However, more detailed functional analyses are required to confirm its role (**Figure 11**).

Are the homologous *LAC* genes from tossa jute (*ColLAC*s) influenced by environmental stresses? GO term assignment indicated that *ColLAC* genes participate in various molecular functions, including oxidoreductase activity (GO:0016491), copper ion binding (GO:0005507), transition metal ion binding (GO:0046914), ion binding (GO:0043167), metal ion binding (GO:0046872), and catalytic activity (GO:0003824) (**Supplementary Figure 2**). These molecular functions may also be associated with plant responses to environmental stress, which warrants further investigation. To investigate this, we applied two types of stress: heavy metal copper and ABA hormone treatment. The rationale for selecting these stresses stems from evidence in the literature suggesting that laccase activity is influenced by copper stress, as copper acts as a cofactor in the enzyme’s function. Additionally, drought, salinity, and cold stress like conditions can be mimicked by ABA hormone hence it is chosen to provide various abiotic stresses in plants (Liu et al., 2017; Sakata et al., 2014; Sharma et al., 2023; Turlapati et al., 2011).

There were some important changes in transcript expression, observed in stress conditions involving copper. These not only revealed the stress-responsive nature of these *ColLAC* genes but also confirmed that the *in-silico*-identified genes are indeed part of the *laccase* family. Under copper stress, the maximum expression of *ColLAC* mRNA transcripts was observed at 8 hours, with fold changes ranging from 8.08 to 41.11 (**Figure 12**). Variability in mRNA transcript levels was noted, with *ColLAC42* showing the highest fold increase (41.11), followed by *ColLAC32* (37.93), *ColLAC34* (21.85), *ColLAC3* (21.47), *ColLAC22* (15.89), *ColLAC46* (10.43), *ColLAC40* (8.09), and *ColLAC38* (8.08). *ColLAC30*, however, did not show significant expression.

Under ABA stress, transcript levels also increased, ranging from 2.98 to 5.97-fold (**Figure 13**). Although the fold changes under ABA stress were lower than those observed under copper stress, they were still statistically significant. Except for *ColLAC3*, *ColLAC38*, and *ColLAC30*, all tested *ColLAC* genes showed peak expression at 4 hours under ABA stress. The fold changes at this time point were as follows, in descending order: *ColLAC22* (5.97), *ColLAC46* (5.58), *ColLAC34* (5.01), *ColLAC32* (3.74), *ColLAC40* (3.57), *ColLAC38* (3.42), *ColLAC42* (3.01), and *ColLAC3* (2.98).

In comparison, the white jute *CcaLAC28* and *CcaLAC32* (homologous to *AtLAC4* and *AtLAC17*, respectively) displayed a delayed response under copper and ABA stresses. For *CcaLAC32*, the highest expression under copper stress occurred at 8 hours, and for *CcaLAC28*, it was observed at 12 hours, in contrast to the 4-hour peak seen in tossa jute. Similarly, under ABA stress, the highest expression of *LAC* in white jute occurred at 24 hours, compared to 4 hours in tossa jute (Parida et al., 2024a). This shift in *LAC* gene expression under abiotic stress may be species-specific. However, the overall fold change ranges for *LAC* gene expression were similar in both species, white and tossa jute.

Gene expression is strongly influenced by upstream cis-acting elements. In order to identify the key cis-acting elements we analysed the 2 kb upstream sequences of all *ColLAC* genes. This search resulted in the discovery of MYB transcription factor (TF) binding sites in several of them (**Supplementary Table 4**). It has been reported that MYB TFs regulate *LAC* expression in various species such as *Phyllostachys edulis* (*PeMYB4.1/20/85.2* regulates *PeLAC20*), Arabidopsis (*AtMYB58* regulates *AtLAC4*), Pennisetum *glaucum* (*PgMYB305* regulates *PgLAC14*) and *Pyrus bretschneideri* (*PbMYB26* regulates *PbLAC4*) (Zhou et al., 2009; Bai et al., 2023). Such studies indicate the potential role of MYB TFs in regulating *ColLAC* gene expression. Other cis-acting elements, in addition to MYB, known to be involved in ABA responsiveness, were identified, these include MYC, W-box, and CAT-box (Fujita et al., 2011). This could explain the varied expression patterns of *ColLAC*s under ABA stress (**Figure 13**).

Gene expression in plants is regulated not only at the transcriptional level but also post-transcriptionally, with microRNAs (miRNAs) playing a crucial role. Although miRNA-mediated gene regulation in jute remains largely unexplored, some studies have previously reported their involvement. For instance, miR-845b and the miR-166 superfamily were found to be expressed in jute under *Macrophomina phaseolina* fungal stress (Dey et al., 2016). *In-silico* analysis of the white jute genome has identified five miRNA superfamilies—miR1536, miR9567-3p, miR4391, miR11300, and miR8689—which may regulate multiple biological functions, including plant growth, cell cycle regulation, organelle synthesis, development, and responses to environmental stresses (Ahmed et al., 2021). Additionally, these miRNAs have the potential to regulate the phenylpropanoid pathway and secondary cell wall formation in white jute. However, the functional validation of these miRNAs remains incomplete, leaving their precise roles in jute unclear. Moreover, miRNAs specifically associated with stem and fibre development in jute are yet to be thoroughly investigated.

Flax, another economically important bast fibre crop, has been more extensively studied in this context. Several miRNAs related to stem and fibre development have been identified in flax, including miR162, miR172, miR395, miR397, and miR530, which show potential roles in stem development (Yu et al., 2021). Additionally, miR390 has been reported to be involved in phloem fibre intrusive growth. Notably, some of these flax miRNA families, particularly miR397, have also been observed in the jute genome, where they are predicted to target the laccase (*LAC*) gene family, a key player in lignification and fibre formation.

The involvement of microRNAs in regulating *LAC* genes has been reported in several plants where miR397 stood out as a well-documented negative regulator of *LAC* genes (Lu et al., 2013). *LAC* expression is regulated by miR397 in various species, including Arabidopsis, Populus, chickpea (*Cicer arietinum*), and rice (Wang et al., 2014; Lu et al., 2008; Sharma et al., 2023; Bakhshi et al., 2016). Here we identified *in-silico* miR397 target sites in the *ColLAC* genes (**Supplementary Table 5**).

A recent study on rapeseed (*Brassica napus*) demonstrated that miR397a regulates the *BnaLAC2* gene, enhancing adaptation to low-temperature stress by modulating lignin remodelling and maintaining ROS (reactive oxygen species) homeostasis (Hussain et al., 2025). This finding suggests that the regulation of *LAC* genes extends beyond lignin biosynthesis, potentially influencing various plant developmental processes and stress response mechanisms. In tossa jute, the role of miRNA-*LAC* interactions remains unexplored. Future functional validation of these regulatory mechanisms will provide deeper insights into how microRNAs fine-tune *LAC* gene expression, thereby shaping both lignification dynamics and stress resilience in this economically significant fibre crop.

A part of the study was also dedicated to the analysis of tissue-specific expression patterns of *ColLAC*s across leaves, stems, and roots (**Figures 8**, **9**). However, what about their cellular expression patterns? An *in-silico* analysis was conducted to investigate *ColLAC* expression in various cellular organelles (**Supplementary Table 2**). The 46 *ColLAC* genes were found to be expressed across nine organelles: chloroplast (15 *ColLAC* genes expressed), cytoplasm (12), vacuoles (10), cytoskeleton (3), extracellular space (2), plastid (1), peroxisome (1), endoplasmic reticulum (1), and nucleus (1). Since many of these are membrane-bound organelles, this suggests that *ColLAC*s may possess structures that enable them to be anchored in membranes. Our findings support this possibility, as transmembrane helices were identified in 45.65% of *ColLAC*s (21 out of 46 genes) (**Supplementary Figure 1**). This insight could be valuable for future structural studies on *ColLAC*s, as currently, very limited structural information is available for LAC proteins in any plant species.

Significant advancements in jute biotechnology have been reported in recent years. These include the development of jute with properties of insect-resistance (Majumder et al., 2018a, 2020b), fungus- and herbicide-resistance (Majumder et al., 2018b), multi-trait jute (Majumder et al., 2024a), and low-lignin jute (Nath et al., 2021). Additionally, cutting-edge gene editing and silencing techniques have been adapted for jute improvement, such as CRISPR/Cas9 technology, virus-induced gene silencing (VIGS), and RNA interference (RNAi) (Jiang et al., 2024; Li et al., 2024; Nath et al., 2021). Stable and efficient gene transformation methods, for both white and tossa jute species, have also been developed (Majumder et al., 2024b). Furthermore, the AtSUC2 promoter, which enables phloem-specific expression of genes in jute fibres, has been successfully validated (Majumder et al., 2020c). These advancements provide a solid groundwork for the further characterization and functional validation of *ColLAC*s genes.

In the near future, overexpression, RNA interference (RNAi), and CRISPR/Cas9-mediated knockout of *ColLAC34* in jute could provide definitive evidence of its role in both lignification and structural development. Additionally, *ColLAC22, ColLAC40*, and *ColLAC46* are likely key regulators of lignin biosynthesis. The first crucial step in this direction was taken in the present study through a comprehensive whole-genome identification of laccase (*ColLAC*) genes in tossa jute, laying the foundation for future functional validation and biotechnological applications.

## 5 Conclusion

Laccase (*LAC*) contributes to lignification as a key player in the final steps of the pathway. The tossa jute genome holds 46 *LAC* genes (*ColLAC*s). Among these 46, nine genes (*ColLAC3, ColLAC22, ColLAC30, ColLAC32, ColLAC34, ColLAC38, ColLAC40, ColLAC42*, and *ColLAC46*) show strong similarity to Arabidopsis *LAC* genes, that are known to be involved in lignin biosynthesis. GO term assignment suggests their likely involvement in the lignification process, and phenylpropanoid pathways. These *ColLAC* genes were analysed and their expression patterns were studied in the plant tissues, at different stages of plant growth, and under various abiotic stresses, such as copper exposure and ABA treatment. Their significant expression levels suggest that these genes play crucial roles in tossa jute’s lignification process. A deeper dive into this wealth of knowledge could open new possibilities in the future, such as targeted regulation of their expression in jute stems to develop low-lignin jute fibres, enabling the production of more diverse biodegradable products, and enhancing the economic value of jute.

## Data Availability

The original contributions presented in the study are included in the article/**Supplementary Material**. Further inquiries can be directed to the corresponding author.

## Supplementary material

Supplementary Figure 1 Detection of transmembrane domains in *ColLAC* proteins.

Supplementary Figure 2 Gene Ontology (GO) term classification of the *ColLAC* gene family.

Supplementary Figure 3 Validation of tissue-specific transcriptomics data through qRT-PCR. Asterisks denote significant differences in *P* values from Tukey’s test, while non-significant values are marked as ‘ns’. The data represent three biological replicates.

Supplementary Table 1 List of Primers.

Supplementary Table 2 Protein features and chromosomal details of the 46 ColLAC members identified in *Corchorus olitorius*.

Supplementary Table 3 List of orthologous and paralogous pairs of *ColLAC* genes.

Supplementary Table 4 List of cis-acting elements in the upstream regions of *ColLAC* genes.

Supplementary Table 5 miRNA targets in *ColLAC* genes.
