# Supplementary figures and images for: Genome-wide analysis of the laccase gene family in tossa jute (Corchorus olitorius): insights into stem development, lignification, and responses to abiotic stress
Source: Front Plant Sci. 2025 May 9;16:1568674. doi: 10.3389/fpls.2025.1568674 (PMC12100665; doi:10.3389/fpls.2025.1568674)

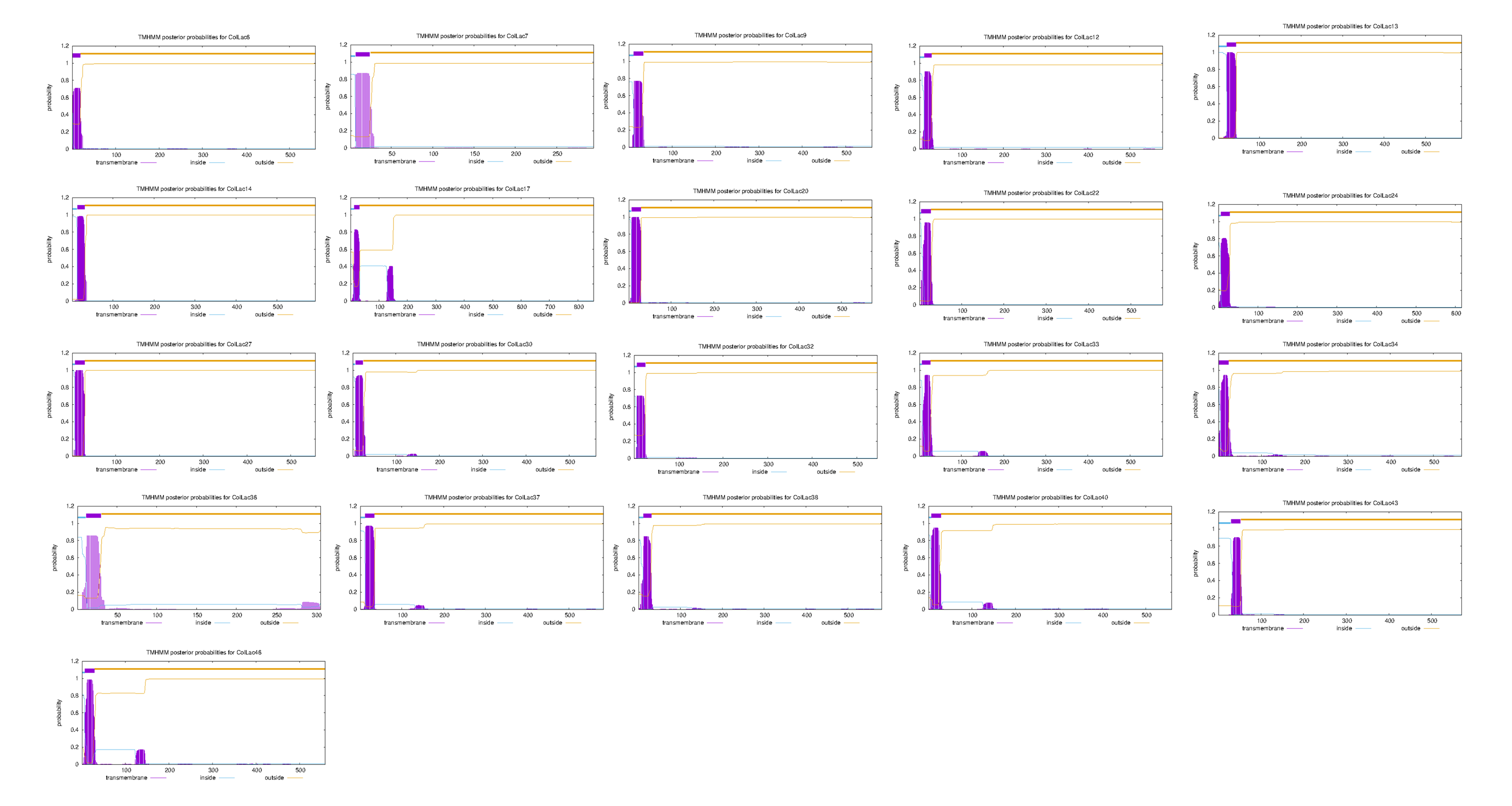

Supplement: Supplementary Figure 1 — Detection of transmembrane domains in ColLAC proteins. [file Image1.tif]

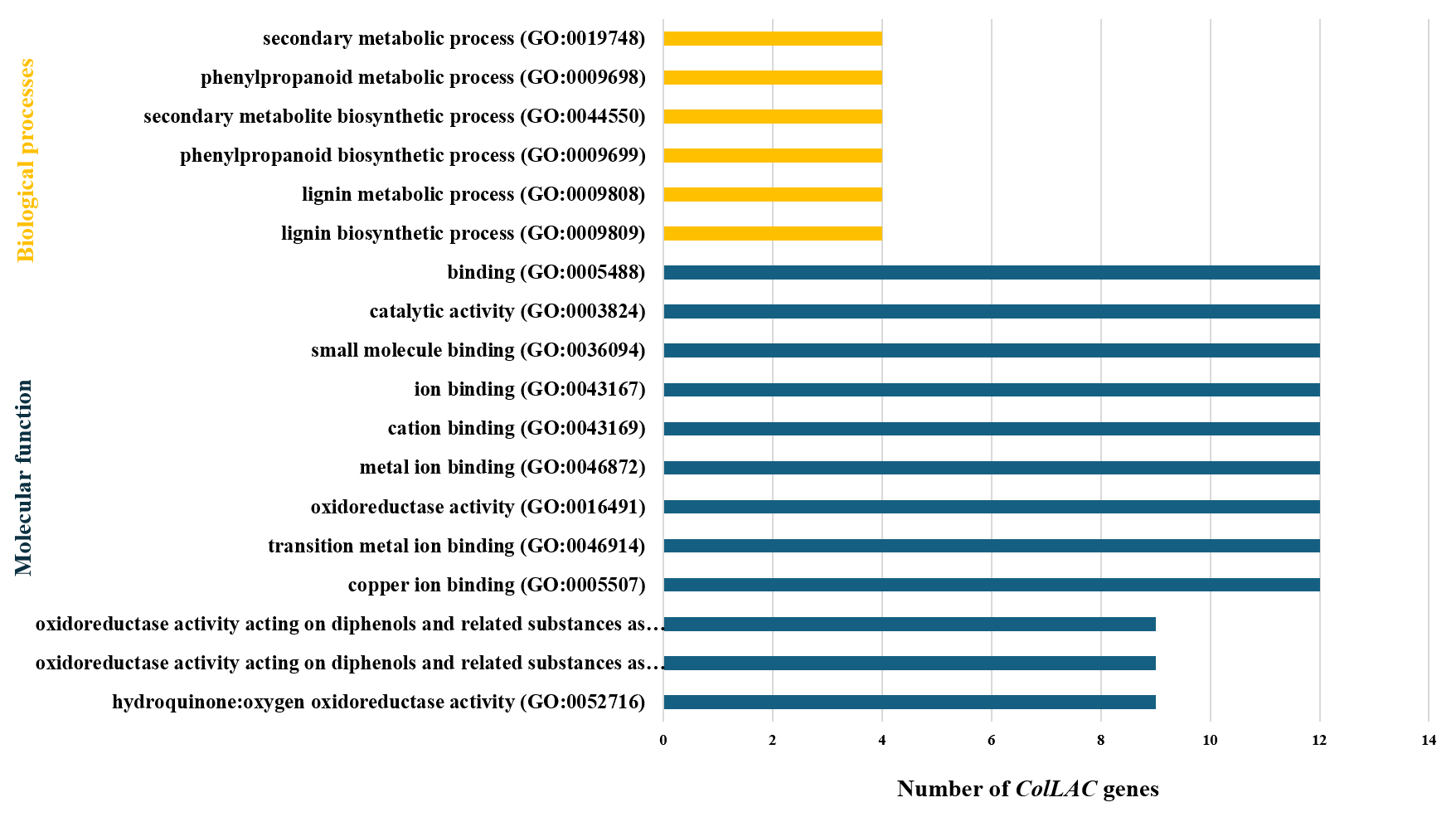

Supplement: Supplementary Figure 2 — Gene Ontology (GO) term classification of the ColLAC gene family. [file Image2.tif]

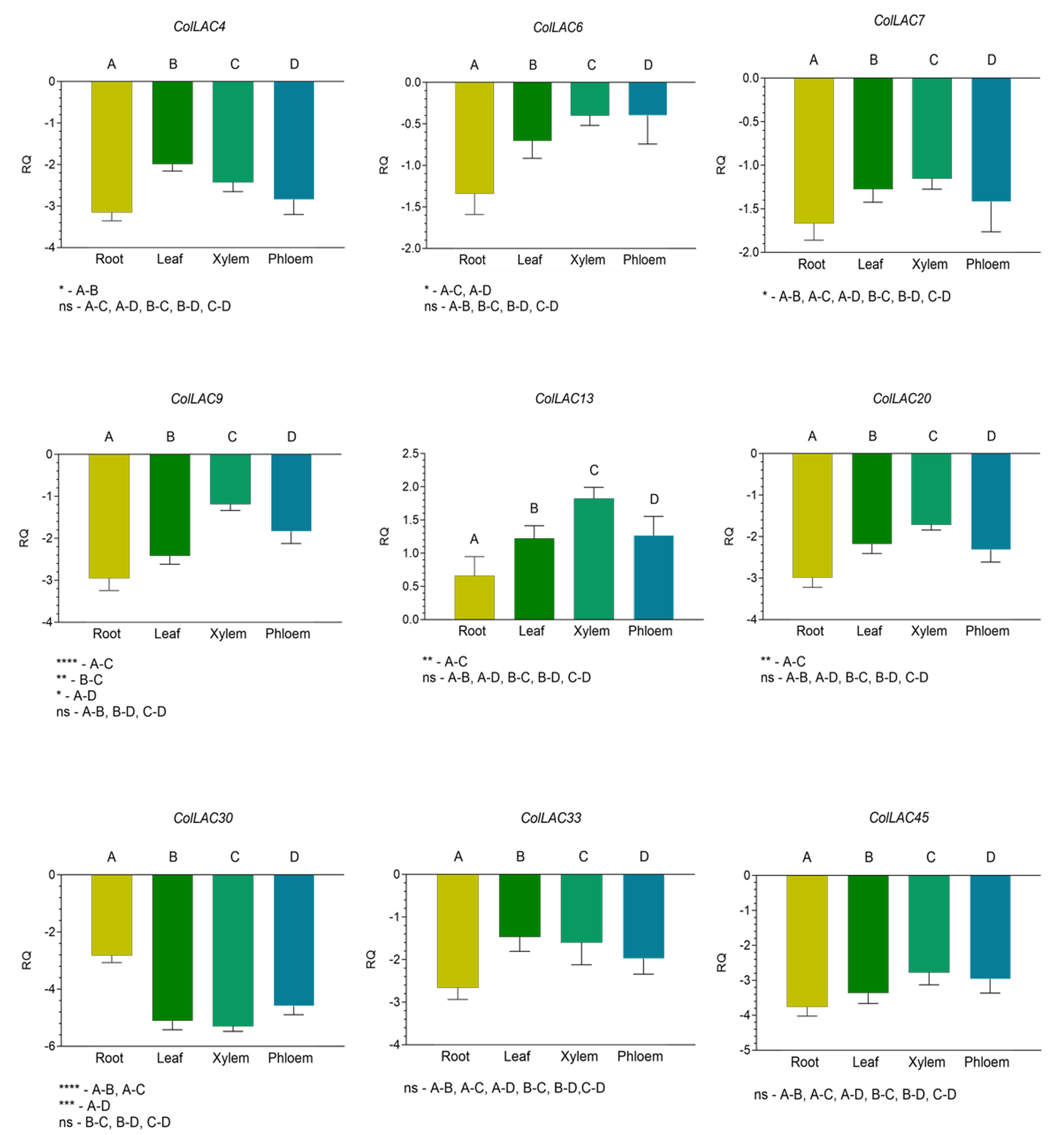

Supplement: Supplementary Figure 3 — Validation of tissue-specific transcriptomics data through qRT-PCR. Asterisks denote significant differences in P values from Tukey’s test, while non-significant values are marked as ‘ns’. The data represent three biological replicates. [file Image3.tif]
